# A Susceptible Cell‐Selective Delivery (SCSD) of mRNA‐Encoded Cas13d Against Influenza Infection

**DOI:** 10.1002/advs.202414651

**Published:** 2025-01-10

**Authors:** Zhuanli Wu, Chengcheng Zhao, Hui Ai, Zhen Wang, Mingyue Chen, Yanli Lyu, Qi Tong, Litao Liu, Honglei Sun, Juan Pu, Ran Zhang, Xiaoxiang Hu, Jinhua Liu, Xiaowei Ma, Yipeng Sun

**Affiliations:** ^1^ National Key Laboratory of Veterinary Public Health and Safety College of Veterinary Medicine China Agricultural University Beijing 100193 China; ^2^ Key Laboratory of Animal Epidemiology of the Ministry of Agriculture and Rural Affairs Key Laboratory for Prevention and Control of Avian Influenza and Other Major Poultry Diseases of the Ministry of Agriculture and Rural Affairs Beijing 100193 China; ^3^ State Key Laboratory of Animal Biotech Breeding College of Biological Sciences China Agricultural University Beijing 100193 China; ^4^ Sanya Institute of China Agricultural University Hainan 572025 China; ^5^ Veterinary Teaching Hospital China Agricultural University Beijing 100193 China; ^6^ Beijing Key Laboratory of Detection Technology for Animal‐Derived Food Safety Beijing 100193 China

**Keywords:** Cas13d mRNA, influenza viruses, susceptible cell‐selective delivery

## Abstract

To bolster the capacity for managing potential infectious diseases in the future, it is critical to develop specific antiviral drugs that can be rapidly designed and delivered precisely. Herein, a CRISPR/Cas13d system for broad‐spectrum targeting of influenza A virus (IAV) from human, avian, and swine sources is designed, incorporating Cas13d mRNA and a tandem CRISPR RNA (crRNA) specific for the highly conserved regions of viral polymerase acidic (PA), nucleoprotein (NP), and matrix (M) gene segments, respectively. Given that the virus targets cells with specific receptors but is not limited to a single organ, a Susceptible Cell Selective Delivery (SCSD) system is developed by modifying a lipid nanoparticle with a peptide mimicking the function of the hemagglutinin of influenza virus to target sialic acid receptors. The SCSD system can precisely deliver an all‐RNA‐based CRISPR/Cas13d system into potentially infected cells. This drug is shown to reduce the viral load in the lungs by 2.37 log_10_ TCID_50_ mL^−1^ and protect 100% of mice from lethal influenza infection. The SCSD‐based CRISPR/Cas13d system shows promise for the flexible and efficient therapy of infections caused by rapidly evolving and novel viruses.

## Graphical abstract

A CRISPR/Cas13d system is designed for broad‐spectrum targeting of IAV, incorporating Cas13d mRNA and a tandem crRNA specific for conserved regions of the viral PA, NP, and M gene segments, respectively. This CRISPR/Cas13d system is delivered by a Susceptible Cell Selective Delivery (SCSD) system which targets sialic acid receptors and shows effective antiviral activity both in vitro and in vivo.

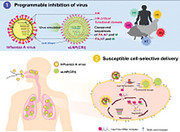

## 1 Introduction

The World Health Organization has issued a global alert regarding “Disease X,” a potential pandemic caused by an unknown pathogen.^[^ ^1^ ^]^ Influenza viruses are considered among the most probable pathogens to cause “Disease X” because of their rapid airborne transmission and high mutation rates. Historically, four influenza pandemics have occurred, with the 1918 Spanish Flu pandemic causing over 50 million deaths.^[^ ^2^ ^]^ Moreover, in recent years, animal influenza viruses have been repeatedly transmitted to humans, usually leading to severe respiratory symptoms and posing a significant pandemic threat.^[^ ^3^, ^4^, ^5^ ^]^ The inevitability of the next influenza pandemic is not a question of “if,” but rather “when.” Furthermore, environmental and climatic changes may expose us to a greater incidence of new and reemergeing viral epidemics. However, the development of novel antiviral drugs requires a long time, so it is difficult to respond rapidly to emerging viruses.

Programmable CRISPR technology offers potential advantages over traditional antiviral drugs: it degrades the viral genome within cells and can thus be rapidly designed based solely on viral sequence information, as well as remaining effective even with delayed administration, which provides swift responses and an extended therapeutic window for emerging viruses. Additionally, it can resist viral escape mutations through targeting conserved sequences and multiple sites, which is a significant advantage in combating highly mutable viruses. Santangelo et al. developed an inhalable CRISPR system with Cas13a mRNA and polymerase basic 1 (PB1)‐targeting CRISPR RNA (crRNA) that can suppress influenza A virus (IAV) in vivo.^[^ ^6^ ^]^ However, Cas13a may have adverse effects owing to its noncanonical trans‐cleavage activity.^[^ ^7^ ^]^ The discovery of Cas13d with higher specificity addressed this issue, exhibiting a smaller molecular weight and greater efficiency.^[^ ^8^ ^]^ Although Cas13d has been reported to effectively inhibit IAV and SARS‐CoV‐2 in vitro,^[^ ^9^ ^]^ its efficiency in vivo remains unclear.

The success of mRNA‐based CRISPR antiviral therapies largely depends on efficient and safe delivery systems that transport mRNA to specific sites. Further, targeted delivery can increase drug utilization and improve efficacy. Nebulized inhalation can directly transport protective carrier‐loaded mRNA to the lungs, but it is less suitable for treating viral infectious diseases because of the significant presence of respiratory mucus, resulting in lower administration efficiency and a shorter half‐life. Alternatively, selective organ‐targeting lipid nanoparticles have been developed for tissue‐specific delivery.^[^ ^10^ ^]^ However, viruses specifically target recognition receptors on host cells, which may be distributed across various organs. The glycoprotein hemagglutinin (HA) of IAV initiates infection by binding to sialic acid receptors (α2,6‐linked and α2,3‐linked sialic acids), which are abundantly expressed in both upper respiratory and lung epithelial cells.^[^ ^11^ ^]^ Thus, cell‐selective delivery will be a more precise and effective therapeutic strategy. Treatment of specific cells minimizes drug exposure to nontarget cells, thereby reducing adverse effects. Until now, this precise design has not yet been reported in antiviral research.

**Figure 1:**
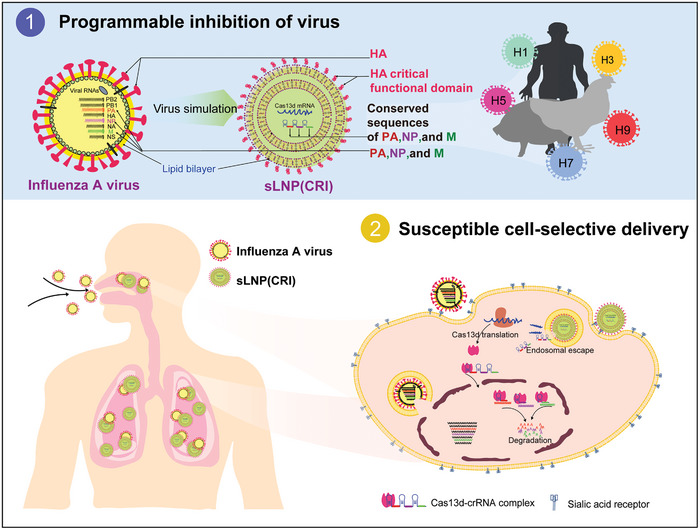
Schematic illustration of the design of sLNP(CRI) and its use in susceptible cell‐selective therapy. A lipid‐based sLNP(CRI) was engineered to mimic the invasion mechanism of IAV by integrating a 30‐amino acid peptide that replicates the function of influenza glycoprotein HA, enabling it to enter susceptible cells. Cas13d mRNA and a tandem crRNA were loaded into sLNP(CRI) to recognize and cleave the conserved sequences of the influenza PA, NP, and M segments. These conserved sequences cover the H1, H3, H5, H7, and H9 subtypes of IAV from human, avian, and swine sources. sLNP(CRI) rapidly reaches the site of IAV infection (upper respiratory tract and lungs) after administration. Subsequently, Cas13d mRNA and tandem crRNA are released into the cytoplasm owing to lysosomal escape capability. Cas13d mRNA is translated into functional Cas13d protein and then forms a Cas13d‐crRNA complex to degrade viral PA/NP/M pre‐mRNA.

In the present study, we constructed a tandem CRISPR/Cas13d system cleaving the conserved regions of the polymerase acidic (PA), nucleoprotein (NP), and matrix (M) gene segments of IAV and delivered this CRISPR/Cas13d system by lipid nanoparticles (LNPs), which target cells with IAV receptors (**Figure** **1**), and we named this drug sLNP(CRI). sLNP(CRI) not only prevents the reduced effectiveness due to viral drug resistance variation but also specifically targets IAV‐susceptible cells. The integration of the programmable CRISPR/Cas13d system and Susceptible Cell‐Selective Delivery (SCSD) provides a platform to rapidly and effectively respond to emerging viruses.

## 2 Results

### 2.1 Computational Design for Cas13d‐Targetable Regions in the IAV Genome

**Figure 2:**
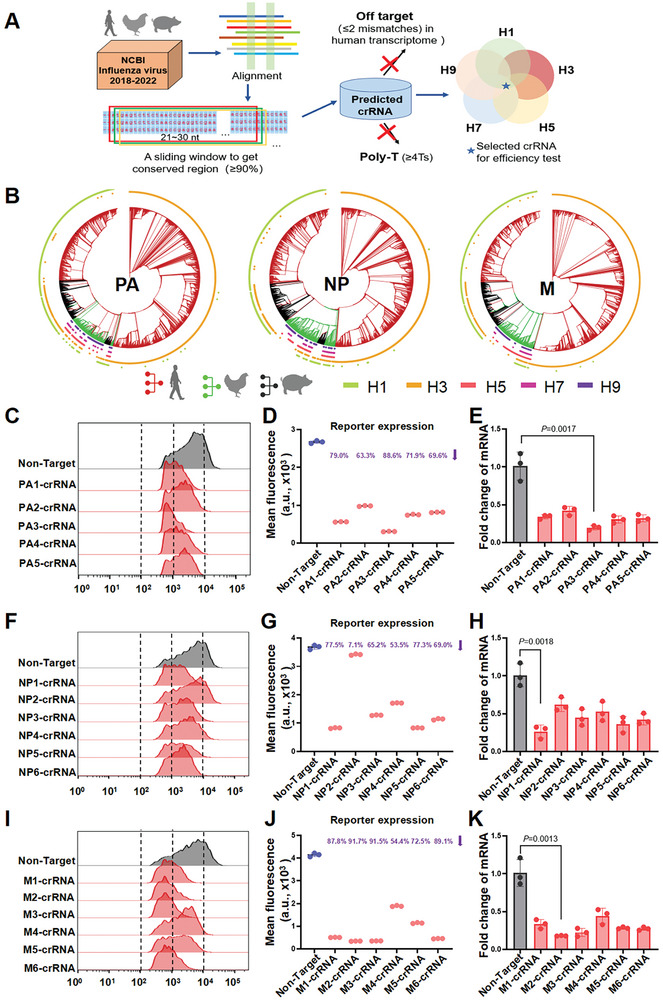
High‐throughput crRNA design for broad‐spectrum targeting of IAV. A) A workflow of Cas13d crRNA analysis for broad‐spectrum targeting of IAV. B) Phylogenetic trees to visualize the evolutionary relationships between H1, H3, H5, H7, and H9 subtype IAV strains isolated from human, avian, and swine sources during the years 2018 to 2022. C–K) Left and middle: mCherry expression as measured by flow cytometry. Right: mRNA abundance of viral sequences as measured by quantitative real‐time PCR. Relative RNA expression was calculated by normalization to the nontarget sample. Data are expressed as the mean ± SD from three biologically independent replicates (*n* = 3). Statistical analysis was performed using unpaired two‐tailed Student's *t*‐tests.

To design crRNAs, we selected the viral genes PA, NP, and M, which have been reported to be relatively conserved and encode proteins acting at different stages of the viral replication cycle.^[^ ^12^, ^13^, ^14^ ^]^ To create specific crRNA sequences for broad‐spectrum targeting of IAV isolated from human, avian, and swine sources, we first performed a bioinformatic analysis (see **Figure** **2**A for the workflow). A total of 86294 published H1, H3, H5, H7, and H9 subtype influenza genomes from these hosts, available in the NCBI database from 2018 to 2022, were aligned (Table S1, Supporting Information displays the number of sequences analyzed for each subtype), and their evolutionary relationships were visualized using phylogenetic trees (Figure 2B). In total, 12594 potential crRNAs, which perfectly matched the conserved regions (covering ≥ 90% sequences in each subtype), were generated according to a previously reported method with some modifications^[^ ^15^ ^]^ (Figure S1A; see Data File 1, Data File 2, and Data File 3 for the crRNA sequences, Supporting Information). After excluding 141 crRNAs with potential off‐target binding sites in the human transcriptome (≤ 2 mismatches) (Figure S1B, Supporting Information; Data File 4) and 1351 crRNAs containing poly‐T sequences (≥ 4 Ts), 11102 crRNAs remained. Among these, 275 crRNAs were shared by at least four subtypes (Figure S1C, Supporting Information).

### 2.2 Screening of crRNAs Targeting IAV

We synthesized 17 crRNA expression vectors, including 5, 6, and 6 crRNAs targeting the PA, NP, and M genes, respectively. Their spacer sequences are detailed in Table S2 (Supporting Information). To screen the effectiveness of the crRNAs, we created three reporters expressing synthesized segments of PA, NP, and M fused to mCherry, each of which was co‐transfected with the Cas13d expression vector and the corresponding crRNA expression vector into 293T cells (Figure S2A, Supporting Information). At 48 h after co‐transfection, RNA was isolated to examine the mRNA transcript abundance of PA, NP, and M, and flow cytometry was performed to examine the levels of mCherry protein expression. Most crRNAs were able to inhibit reporter expression to a certain extent compared with the nontarget control (Figure 2C,F,I), and one crRNA targeting the 5ʹ region of the M segment (M2‐crRNA) was remarkably effective and able to inhibit mCherry by 91.7% (Figure 2J). Two crRNAs (PA3‐crRNA and NP1‐crRNA) were able to substantially repress mCherry, causing 88.6% and 77.5% decreases in expression, respectively (Figure 2D,G). The results of quantitative real‐time PCR analysis were consistent at the RNA level, with M2‐crRNA, NP1‐crRNA, and PA3‐crRNA inhibiting their corresponding reporter mRNA abundance by 82.0%, 73.6%, and 80.4%, respectively (Figure 2E,H,K). To verify that the reporter‐validated crRNAs effectively targeted IAV, 24 h after co‐transfection of the crRNA and Cas13d expression vectors, human lung epithelial (A549) cells were infected with A/Puerto Rico/8/1934 (PR8, H1N1) at a multiplicity of infection (MOI) of 0.01. At 24 h post‐infection (hpi), the virus titer was measured by a TCID_50_ assay and indicated a reduction in virus titer by 1.00–1.17 log_10_ TCID_50_ mL^−1^ (Figure S2B, Supporting Information).

We constructed a tandem crRNA expression vector that co‐expressed PA3‐crRNA, NP1‐crRNA, and M2‐crRNA (Figure S2C, Supporting Information), and Figure S2B (Supporting Information) shows that this tandem crRNA reduced the virus titer by 1.25 log_10_ TCID_50_ mL^−1^. Furthermore, we analyzed the major influenza strains prevalent in 2023 targeted by this tandem crRNA, and 93.29%, 98.42%, and 86.57% of strains were covered by PA3‐crRNA, NP1‐crRNA, and M2‐crRNA, respectively, with perfect matches (Figure S2D, Supporting Information). Cas13d is able to effectively cleave its target sequence even if it harbors a single mismatch, meaning that all of the analyzed strains could be targeted.

### 2.3 Design and Characterization of sLNP(CRI)

**Figure 3:**
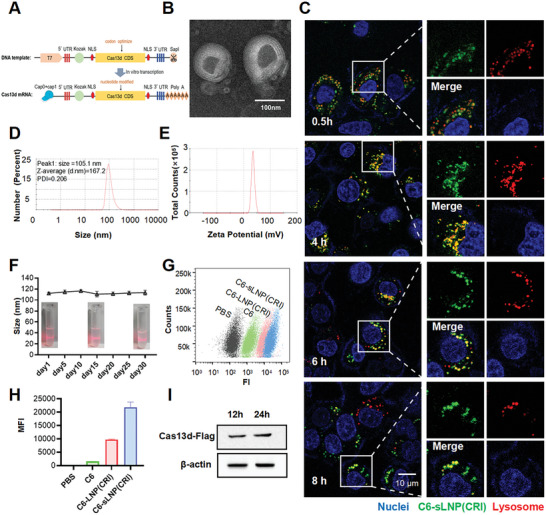
Preparation and characterization of sLNP(CRI). A) Schematic representation of Cas13d mRNA modification and synthesis. B) TEM images of sLNP(CRI). C) Confocal images showing the localization of C6‐labeled sLNP(CRI) relative to lysosomes after incubation with A549 cells for 0.5, 4, 6, and 8 h. The lysosomes were stained with Lysotracker Red DND‐99. D,E) Size distribution and zeta potential of sLNP(CRI). F) Changes in the diameter of sLNP(CRI) over 30 days. G) Quantitative analysis of free C6, C6‐LNP(CRI), and C6‐sLNP(CRI) taken up by A549 cells using flow cytometry. H) Mean fluorescence intensity (MFI) of A549 cells after treatment with free C6, C6‐LNP(CRI), and C6‐sLNP(CRI) for 4 h. I) Western blot analysis showing Cas13d protein expression.

The Cas13d mRNA and tandem crRNA were acquired through transcription in vitro. Codon optimization and uridine modification were performed to improve the efficiency of mRNA expression and decrease immunogenicity (**Figure** **3**A and Data File 5). Increased mRNA expression and reduced immunogenicity were demonstrated in A549 cells and the lungs of mice (Figures S3 and S4, Supporting Information). To date, LNPs are the only RNA delivery system approved by the FDA for clinical use. To enable the LNPs to specifically bind to IAV‐susceptible cells, we conjugated a peptide that mimics the function of influenza glycoprotein HA and recognizes a pattern of sugar chains in the receptor to 1,2‐distearoyl‐sn‐glycero‐3‐phosphoethanolamine‐N‐[methoxy(polyethylene glycol)‐3400] (DSPE‐PEG3400).^[^ ^16^, ^17^ ^]^ Ionizable lipid 1,2‐dioleoyl‐3‐trimethylammonium propane (DOTAP), which significantly enhances endosomal escape efficiency,^[^ ^18^ ^]^ was added into our design. Briefly, sLNP(CRI) was formulated mainly with 1,2‐dioleoyl‐sn‐glycero‐3‐phosphocholine (DOPC), and prepared by the thin‐film hydration method. Additionally, we synthesized LNP(CRI), which lacks the HA critical functional domain, to serve as a control group not treated with SCSD.

The complexation capacity of RNA (Cas13d mRNA and crRNA) by sLNP(CRI) was evaluated by a gel retardation assay as previously reported.^[^ ^19^, ^20^, ^21^ ^]^ As the nitrogen/phosphorus (N/P) ratio increased, the amount of unbound RNA decreased until a ratio of 4/1 was reached at which all of the RNA was fully loaded (Figure S5, Supporting Information). At this N/P ratio, the mass ratio of RNA to lipid was 1:428. Transmission electron microscopy (TEM) observations revealed that sLNP(CRI) had a typical spherical structure (Figure 3B). The average size and surface charge, both measured using a Zetasizer Nano ZS, were 100 ± 20 nm (Figure 3D) and 37.6 mV (Figure 3E), respectively. In addition, sLNP(CRI) remained stable for at least a month without a visible change in size (Figure 3F). We next explored the intracellular kinetic behavior of sLNP(CRI). A549 cells treated with coumarin‐6 (C6, green)‐labeled sLNP(CRI) showed a notable rightward shift in flow cytometry (Figure 3G), and the mean fluorescence intensity was 14.09 times higher than for cells with free C6, and 2.24 times higher than C6‐labeled LNP(CRI) (Figure 3H). As shown in Figure 3C, most of the sLNP(CRI) colocalized with lysosomes at the 4 h time point. Interestingly, an increase in green fluorescence was detected in the cytosol after 6 h, indicating that sLNP(CRI) possesses the ability to escape from endosomes, which is essential for successful Cas13d mRNA translation. Furthermore, we demonstrated that sLNP(CRI) enables strong expression of mRNA‐encoded Cas13d both in vitro and in vivo (Figure 3I and Figure S3, Supporting Information), and crRNA was stable for at least 12 h in mice (Figure S6, Supporting Information), enabling the subsequent process of crRNA binding to Cas13d protein.

### 2.4 Susceptible Cell‐Selective Delivery

**Figure 4:**
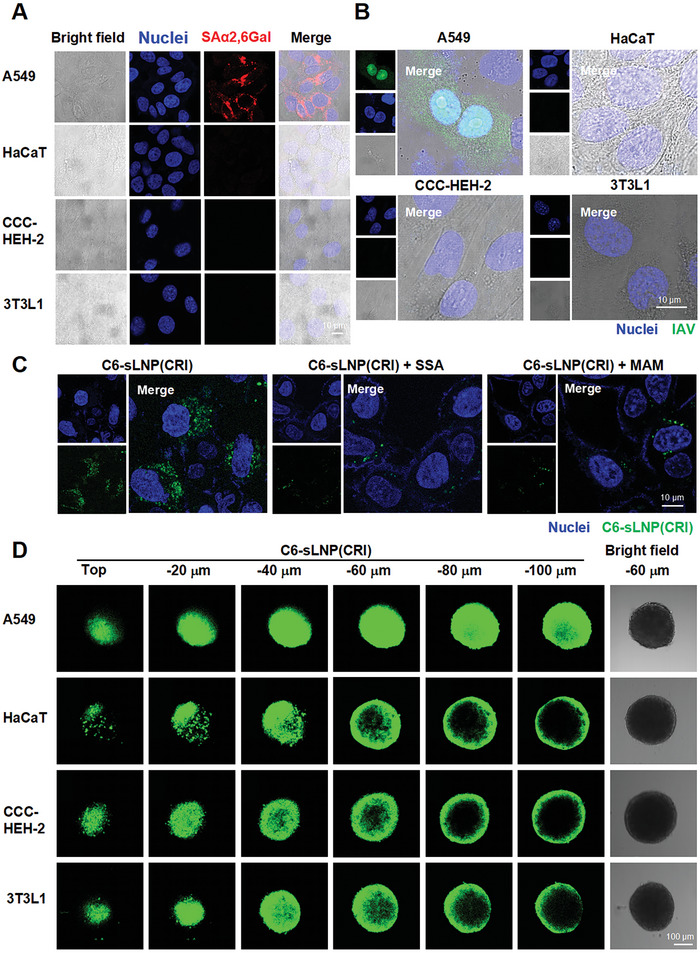
Susceptible cell‐selective delivery of sLNP(CRI). A) Identification of sialic acid receptor expression in A549, HaCaT, CCC‐HEH‐2, and 3T3L1 cells. Biotinylated SSA bound to SAα2,6 Gal and was then detected using Dylight 549‐labeled streptavidin. B) The ability of IAV to infect A549, HaCaT, CCC‐HEH‐2, and 3T3L1 cells. C) Inhibition of the cellular uptake of sLNP(CRI) in a sialic acid receptor competition binding assay. The A549 cells were preincubated with 10 µm lectins (with high affinity for sialic acid α2,6‐galactose or sialic acid α2,3‐galactose), and the cells were subsequently incubated with C6‐labeled sLNP(CRI) for 2 h. Nuclei were stained with Hoechst 33342. D) The specific binding and penetration of sLNP(CRI) in 3D spheroids of A549, HaCaT, CCC‐HEH‐2, and 3T3L1 cells. After incubating for 4 h, confocal microscopy images were obtained by scanning the spheroids from top to bottom at a depth of 20 µm per image.

IAV infection is initiated by interactions between viral HA protein and sialic acid receptors on target cells. We showed that A549 cells that express α2,6‐linked and α2,3‐linked sialic acids were able to be infected by PR8 virus, whereas human immortalized keratinocytes (HaCaT) cells, human embryonic cardiac tissue‐derived (CCC‐HEH‐2) cells, and mouse embryo fibroblasts (3T3L1), which do not possess IAV receptor, cannot be infected by PR8 virus (**Figures** **4**A,B and S7, Supporting Information). To demonstrate the specific binding of sLNP(CRI) to IAV receptors, we performed a receptor competition binding assay, and the cellular uptake of C6‐labeled sLNP(CRI) by A549 cells was evaluated in the presence or absence of lectins. *Sambucus sieboldiana agglutinin* (SSA) and *Maackia amurensis agglutinin* (MAM) are lectins with high affinity for sialic acid α2,6‐galactose (SAα2,6 Gal) and sialic acid α2,3‐galactose (SAα2,3 Gal), respectively. We observed that the cellular uptake of sLNP(CRI) was significantly blocked in the presence of 10 µm MAM or SSA (Figure 4C).

To further evaluate the specific delivery of sLNP(CRI) in IAV‐susceptible cells, we constructed 3D spheroids of A549, HaCaT, CCC‐HEH‐2, or 3T3L1 cells, respectively. As shown in Figure 4D, C6‐labeled sLNP(CRI) penetrated the deep inner region of the spheroid formed from A549 cells, but was only distributed in the outer region, and the first few layers of the spheroids formed from HaCaT, CCC‐HEH‐2, or 3T3L1 cells. This indicated that the specific binding of sLNP(CRI) to sialic acid receptors enhanced receptor‐mediated endocytosis, thereby increasing its ability to penetrate into the inner part of the 3D spheroids.

### 2.5 Inhibition of IAV In Vitro

**Figure 5:**
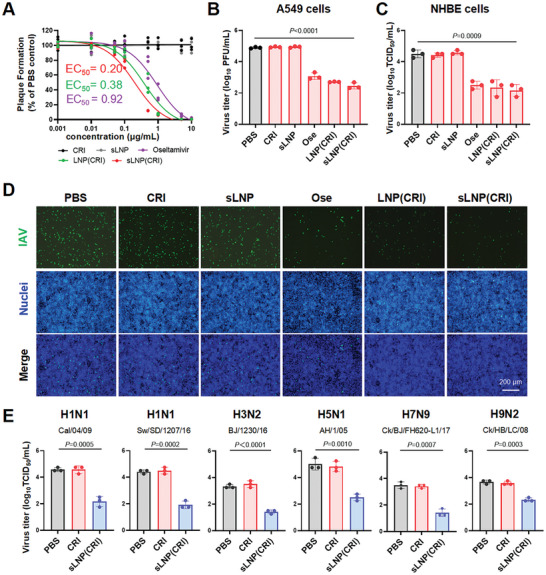
Antiviral activity of sLNP(CRI) in vitro. A) Effects of the indicated drug on plaque formation by PR8 virus were determined at different concentrations. CRI: free crRNA and Cas13d mRNA. sLNP group: the lipid concentration is equivalent to sLNP(CRI). B) Reduction in virus titers at 10 µg mL^−1^ of the indicated drug in A549 cells. Oseltamivir: 10 µg mL^−1^; CRI, LNP(CRI), or sLNP(CRI): 10 µg mL^−1^ of RNA. C) NHBE cells were infected with PR8 virus and treated with 10 µg mL^−1^ of the indicated drug. Virus titers in supernatants were determined by a TCID_50_ assay. D) A549 cells were infected with PR8 virus and treated with 10 µg mL^−1^ of the indicated drug. Cells were stained with anti‐NP (green) and DAPI (blue) at 36 hpi. E) The effectiveness of sLNP(CRI) against various subtypes of influenza viruses. Data are expressed as the mean ± SD from three biologically independent replicates (*n* = 3). Statistical analysis was performed using unpaired two‐tailed Student's *t*‐tests.

To evaluate the inhibition of IAV by sLNP(CRI), we assessed the antiviral efficacy of sLNP(CRI) under a range of RNA concentrations from 0.001 to 10 µg mL^−1^. A549 cells were infected with PR8 virus at an MOI of 0.01, and a plaque assay was employed to quantify infectious virions in the supernatant at 36 hpi. As shown in **Figure** **5**A, free Cas13d mRNA and crRNA (CRI group) did not show any inhibition of the virus. The anti‐influenza effect of sLNP(CRI), the EC_50_ value of which was 0.20 µg mL^−1^ (95% CI of 0.15 to 0.28 µg mL^−1^), was superior to that of clinically used Oseltamivir (0.92 µg mL^−1^, 95% CI of 0.60 to 1.43 µg mL^−1^). Additionally, sLNP(CRI) showed significantly more efficient antiviral activity compared with Oseltamivir at drug concentrations of 0.1–1 µg mL^−1^. Notably, the viral titer was reduced by 2.44 log_10_ PFU mL^−1^ when the concentration of sLNP(CRI) reached 10 µg mL^−1^ (Figure 5B). An immunofluorescence assay demonstrated a significant reduction in viral replication when treated with sLNP(CRI) (10 µg mL^−1^) in A549 cells (Figure 5D). We further examined the effectiveness of sLNP(CRI) in primary normal human bronchial epithelial (NHBE) cells at a drug concentration of 10 µg mL^−1^. NHBE cells were infected with PR8 virus at an MOI of 0.01, and sLNP(CRI) reduced the viral titer by 2.34 log_10_ TCID_50_ mL^−1^ (Figure 5C).

To verify the broad‐spectrum anti‐influenza effect of sLNP(CRI), we examined the effectiveness of sLNP(CRI) (10 µg mL^−1^) against various subtypes of IAV, including human, avian, and swine influenza strains, showing a reduction in viral titer of 1.33–2.50 log_10_ TCID_50_ mL^−1^ (Figure 5E).

### 2.6 Safety and Biodistribution

**Figure 6:**
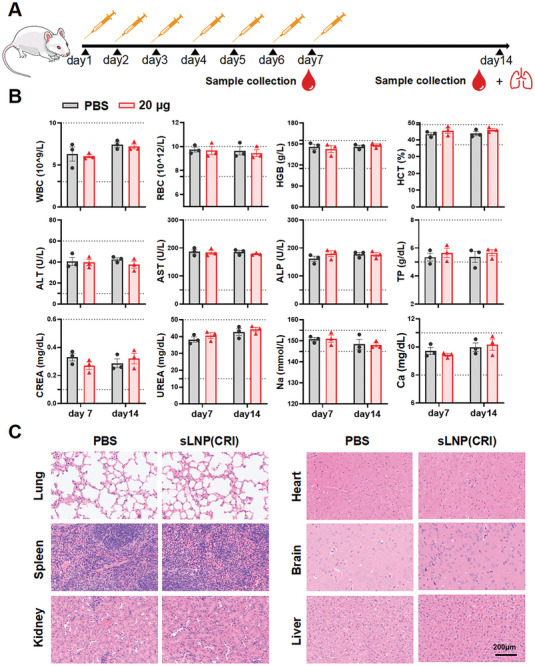
In vivo safety of sLNP(CRI). A) Schematic of the safety evaluation process in vivo. B) Hematology evaluation (WBC, white blood cell; RBC, red blood cell; HCT, hematocrit; HGB, hemoglobin), and blood biochemistry analysis (ALT, alanine transferase; AST, aspartate transferase; TP, total protein; ALP, alkaline phosphatase; CREA, creatinine; UREA, blood urea nitrogen; Na; Ca) of mice on day 7 and day 14 after injection of sLNP(CRI) with 20 µg RNA; the normal range is marked by a dashed line. C) H&E staining of the brain, liver, heart, lung, kidney, and spleen of mice from different groups on day 14. Data are expressed as the mean ± SD from three biologically independent replicates (*n* = 3). Statistical analysis was performed using unpaired two‐tailed Student's *t*‐tests.

A hemolysis assay was performed and revealed no hemolytic toxicity even after treatment with the highest concentration of sLNP (320 µg mL^−1^) (Figure S8, Supporting Information). Additionally, all red blood cells exhibited a uniform dispersion and maintained a favorable round morphology after sLNP (320 µg mL^−1^) treatment for 3 h (Figure S8, Supporting Information). Furthermore, the sensing of influenza RNA generated by Cas13d cleavage did not induce type I interferons (Figure S9, Supporting Information). Continuous administration of sLNP(CRI) (20 µg RNA) for 7 days in mice was conducted to assess safety (see **Figure** **6**A for the timeline). The analysis of hematology (WBC, white blood cell; RBC, red blood cell; HCT, hematocrit; HGB, hemoglobin) in both the PBS and sLNP(CRI) groups revealed no significant differences (Figure 6B). Furthermore, biochemical analyses of hepatic (ALT, alanine transferase; AST, aspartate transferase; TP, total protein; ALP, alkaline phosphatase) and renal (CREA, creatinine; UREA, blood urea nitrogen; Na; Ca) function indicated that all data remained within the normal ranges (marked by dashed lines) (Figure 6B). Consistently, the histopathological structure of the principal organs (e.g., heart, liver, spleen, lung, brain, and kidney) remained normal without apparent inflammatory cell invasion (Figure 6C). Additionally, anti‐Cas13d immunoglobulin G (IgG) was not evoked by repeated sLNP(CRI) injection (Figure S10, Supporting Information). Therefore, sLNP(CRI) was considered relatively safe for clinical treatment.

**Figure 7:**
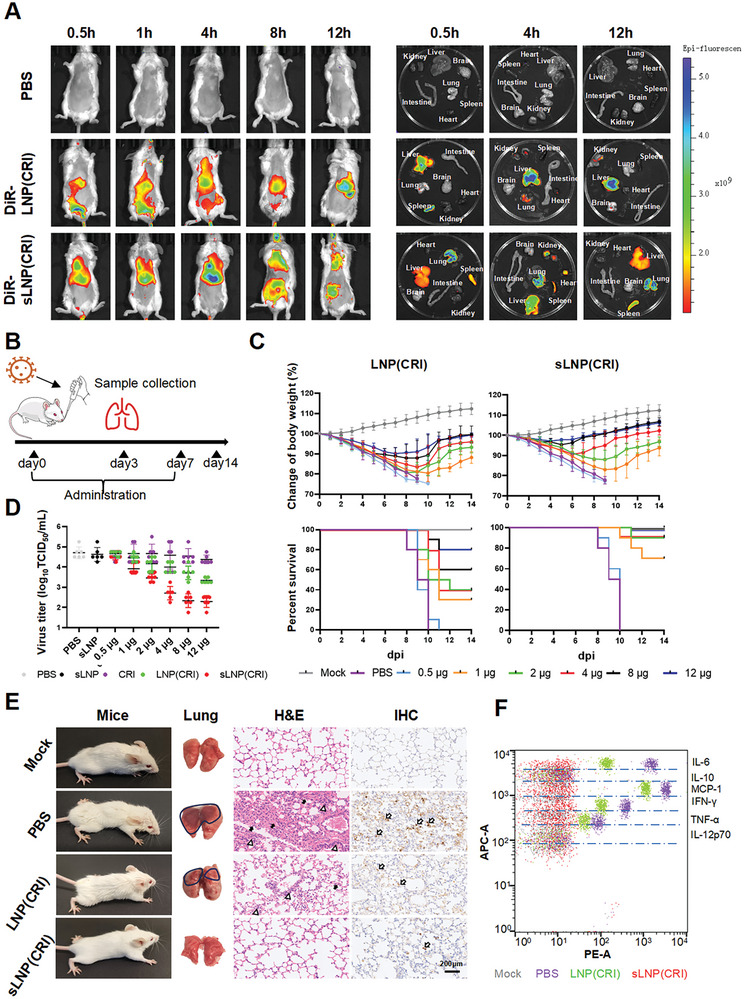
Biodistribution and antiviral activity of sLNP(CRI) in vivo. A) In vivo and ex vivo IVIS imaging for verifying the distribution and respiratory (lung and nasopharynx) targeting ability of sLNP(CRI). Mice were administered either DiR‐labeled LNP(CRI) or DiR‐labeled sLNP(CRI) via the tail vein at a dose of 20 µg DiR per mouse. B) Timeline of the lethal mouse model of influenza. C) The body weights (*n* = 10) and survival rates of the mice. Mice were inoculated with PR8 virus on day 0, and their body weights were recorded for 14 days. When weight loss accounted for more than 25% of the initial body weight, the mouse was euthanized. D) Viral titers in the lung homogenates were quantified as the TCID_50_ value at 3 dpi (*n* = 6). E) Representative photographs of mice and mice lungs administered different treatments. H&E staining of lungs: thick solid arrow indicates alveolar wall thickening, and a black hollow triangle indicates immune cell infiltration. Immunohistochemical staining with anti‐NP monoclonal antibodies shows the positive detection rate of viral NP protein; thick open arrows indicate positive areas. F) Levels of inflammatory cytokines in the lungs of mice were measured. Samples were collected at 3 dpi. Statistical analysis was performed using unpaired two‐tailed Student's *t*‐tests.

Before evaluating the therapeutic efficacy in vivo, we assessed the biodistribution and targeting ability of sLNP(CRI) labeled with DiR using an in vivo imaging system (IVIS), as compared with LNP(CRI). The time‐dependent distribution of DiR in the main organs was monitored both in vivo and ex vivo. Fluorescence signals in the DiR‐LNP(CRI) group were found to be predominantly accumulated in the liver at all time points examined. By contrast, DiR‐labeled sLNP(CRI) showed strong and rapid uptake into the lungs at 0.5 h post‐injection, along with weak accumulation in the liver (**Figure** **7**A). The intense signal remained in the lungs for 12 h, and a strong signal was observed in the nasopharyngeal region at 8 h post‐injection, demonstrating the excellent IAV susceptible cell‐targeting ability of sLNP(CRI). We noticed that the fluorescence signals in all tissues attenuated at 24 h post‐injection and became undetectable at 36 h post‐injection (Figure S11, Supporting Information).

### 2.7 Treatment of Influenza Infection in Mice

We further investigated whether sLNP(CRI) could protect mice from lethal IAV infection. In brief, mice were inoculated intranasally with 100 TCID_50_ PR8 to induce lethal pneumonia and received sLNP(CRI) or LNP(CRI) containing different doses via tail vein administration daily until day 7. The timeline for administration is illustrated in Figure 7B. The viral load in the lungs was evaluated at 3 days post‐infection (dpi), and the symptoms and weight changes of mice were observed for 14 days. As shown in Figure 7C, the administration of LNP(CRI) and sLNP(CRI) to IAV‐challenged mice significantly improved survival rates in a dose‐dependent manner. Notably, 100% of the mice treated with 8 or 12 µg RNA by sLNP(CRI) survived, while 60% or 80% of the mice treated with the same doses by LNP(CRI) survived, respectively. By contrast, all of the mice treated with PBS died within 10 dpi. We further demonstrated that, at the optimal dose of 8 µg RNA, sLNP(CRI) reduced the virus titer by 2.37 log_10_ TCID_50_ mL^−1^, compared with a reduction of 1.00 log_10_ TCID_50_ mL^−1^ by LNP(CRI) (Figure 7D). Mice receiving sLNP(CRI) with 8 µg RNA maintained normal coat hair, whereas mice in the PBS and LNP(CRI) (8 µg RNA) groups exhibited messy coat hair, indicating that SCSD significantly enhances the in vivo antiviral efficacy of the CRISPR system (Figure 7E). Hematoxylin and eosin (H&E) staining showed that mice in the PBS group displayed thickening of the alveolar wall and obvious inflammatory cell infiltration, and the LNP(CRI) group exhibited moderate levels of inflammatory damage. Comparatively, mice treated with sLNP(CRI) showed normal lung tissue architecture (Figure 7E). Immunohistochemical staining revealed the minimal presence of IAV antigens in the sLNP(CRI) group, whereas a large quantity of IAV antigens (brown color) was observed in the PBS group and a slightly reduced amount was observed in the LNP(CRI) group (Figure 7E). These observations further demonstrate that the viruses were efficiently inhibited by sLNP(CRI). Besides, controlling the cytokine storm is essential for decreasing mortality associated with IAV‐induced pneumonia. As shown in Figure 7F, we observed that the expression levels of several inflammatory cytokines in the sLNP(CRI) group with 8 µg RNA, including IL‐6, MCP‐1, IFN‐γ, and TNF‐α, were substantially reduced to levels similar to those in the mock group.

**Figure 8:**
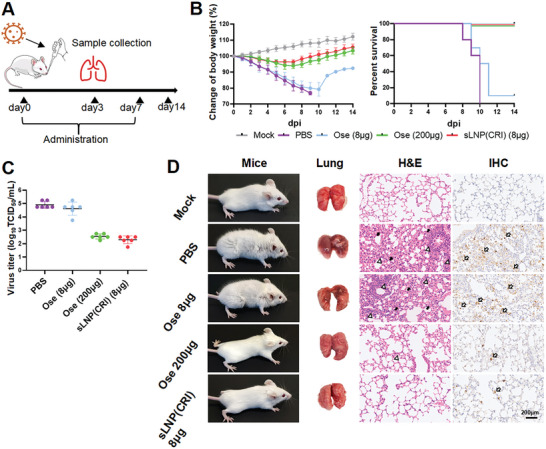
Antiviral activity of sLNP(CRI) and Oseltamivir in vivo. A) The timeline for administration and sample collection. B) The body weights (*n* = 10) and survival rates of the mice. Mice were inoculated with PR8 virus on day 0, and their body weights were recorded for 14 days. C) Viral titers (*n* = 6) in the lung. D) Representative photographs of mice and mice lungs administered different treatments. H&E staining of lungs: thick solid arrows indicate alveolar wall thickening and black hollow triangles indicate immune cell infiltration. Immunohistochemical staining with anti‐NP monoclonal antibodies shows the positive detection rate of viral NP protein; thick open arrows indicate positive areas. Statistical analysis was performed using unpaired two‐tailed Student's *t*‐tests.

**Figure 9:**
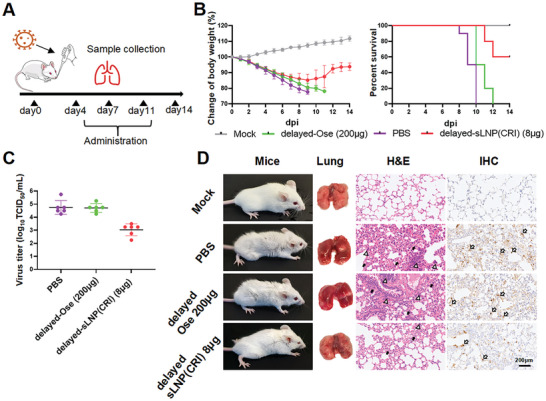
Antiviral activity with delayed administration of sLNP(CRI) and Oseltamivir in vivo. A) The timeline for delayed administration and sample collection. B) The body weights (*n* = 10) and survival rates of the mice. Mice were inoculated with PR8 virus on day 0, and their body weights were recorded for 14 days. C) Viral titers (*n* = 6) in the lung. D) Representative photographs of mice and mice lungs following delayed administration. H&E staining of lungs: thick solid arrows indicate alveolar wall thickening and black hollow triangles indicate immune cell infiltration. Immunohistochemical staining with anti‐NP monoclonal antibodies shows the positive detection rate of viral NP protein; thick open arrows indicate positive areas. Statistical analysis was performed using unpaired two‐tailed Student's *t*‐tests.

Additionally, we compared the antiviral efficacy of sLNP(CRI) (8 µg per mouse) and Oseltamivir (an equivalent dose to sLNP(CRI) of 8 µg per mouse, or a clinically recommended dose of 200 µg per mouse). **Figure** **8** shows that Oseltamivir was unable to significantly inhibit viral replication at 8 µg per mouse, and the dose required to effectively protect mice from mortality was 25 times higher than that of sLNP(CRI). We next evaluated the therapeutic effect of delayed administration of sLNP(CRI) and Oseltamivir (**Figure** **9**A). The sLNP(CRI)‐treated group exhibited relatively less weight loss and a lower viral load, and body weight eventually recovered, with a survival rate of 60%, whereas all mice in the Oseltamivir‐treated group showed severe weight loss and died (Figure 9B–D). Thus, sLNP(CRI) exhibited a high efficacy whether administered during the early phase or late phase of IAV infection. Additionally, we showed that the targeting ability and efficacy of sLNP(CRI) were not affected by anti‐HA antibodies (Figure S12, Supporting Information).

## 3 Discussion

With changes in the climate and environment, we may confront more emerging and re‐emerging viral epidemics. Viruses characterized by their high mutation rate and airborne transmission, such as influenza and coronaviruses, continually cause pandemics. However, antiviral research has been based on limited strategies, such as structure‐based drug design^[^ ^22^, ^23^, ^24^ ^]^ and mechanism‐based drug design.^[^ ^25^, ^26^, ^27^ ^]^ These approaches require long‐term research investment and may not effectively respond to the rapid variation of viruses. When faced with an emerging virus, the initial information we can obtain consists of viral sequences. The next‐generation sequencing technique enabled researchers to obtain the complete viral genome within two weeks of the first emergence of the virus during the COVID‐19 pandemic.^[^ ^28^ ^]^ The programmable CRISPR/Cas13 system relies solely on the knowledge gained from viral genomic sequences and is capable of degrading the viral genome, and thus holds promise as an effective antiviral strategy for the rapid response to emerging viruses. Additionally, traditional drugs targeting viral proteins usually act at a specific stage of the viral replication cycle; for example, the specific anti‐influenza drug Oseltamivir can only inhibit NA protein activity when the virus buds and becomes ineffective once the virus is actively replicating within cells. By contrast, CRISPR technology allows for drug administration at any stage during infection to directly degrade the viral genome.

LNPs have been demonstrated to be effective and safe for delivering an all‐RNA‐based CRISPR system. Hotta et al. reported an LNP‐based approach capable of delivering Cas9 mRNA and sgRNA into skeletal muscles via intramuscular injection.^[^ ^29^ ^]^ Xu et al. achieved an efficient knockdown of the Angptl3 gene in the liver through Cas9 mRNA‐loaded LNPs.^[^ ^30^ ^]^ However, most previously reported LNPs lack targeting capabilities, which is crucial for achieving effective therapy at lower doses. Additionally, organ‐selective targeting is not suitable for viral infections, since cells susceptible to viruses may be distributed across multiple organs. IAV, for example, can replicate extensively throughout the respiratory system, including the nasopharynx and lungs. Notably, seasonal influenza viruses predominantly replicate in the upper respiratory tract; whereas during influenza pandemics, such as the 1918 Spanish flu pandemic and the 2009 H1N1 pandemic, a significant number of cases evolved into primary viral pneumonia. Thus, a single organ‐targeted therapeutic is inadequate against IAV infections, underscoring the necessity for infected cell‐selective treatment. To this end, we conjugated a sugar chain targeting ligand in peptide form to the LNP; this peptide has been proven to bind to terminal sialic acid chains of receptors.^[^ ^17^ ^]^ In mice, we observed that sLNP(CRI) with SCSD accumulated in the nasopharynx and lungs, aligning with the tissues of influenza virus infection. Compared with mice treated with LNP(CRI) without SCSD, the survival rate of mice treated with sLNP(CRI) with SCSD was increased, with a marked reduction in viral load and inflammatory levels in the lungs. Overall, our SCSD‐based CRISPR/Cas13d system reduced the viral load in the lungs by 2.37 TCID_50_ mL^−1^, protecting mice against death during lethal influenza infection.

In conclusion, the present study highlights the significant potential of using SCSD combined with CRISPR/Cas13d technology as a flexible, rapid‐response tool against emerging viruses, providing a promising avenue for the successful in vivo application of CRISPR.

## 4 Experimental Section

### High‐Throughput crRNA Design

All of the influenza sequences obtained from 2018 to 2022 were retrieved from the NCBI database (www.ncbi.nlm.nih.gov), and the sequences were aligned by Multiple Alignments using Fast Fourier Transform (MAFFT version 7). To obtain a greater quantity and broader coverage of crRNAs, the code reported by Qi et al. was modified.^[^ ^15^ ^]^ A 21‐ to 30‐nucleotide sliding window was passed over the alignment, and if a sequence of this length appeared in ≥ 90% of aligned sequences, it was added to the crRNA set. To remove crRNAs with off‐target sites in the human transcriptome, Bowtie2 was used to recognize crRNAs that align to the human transcriptome with ≤ 2 mismatches, and these crRNAs as well as crRNAs with a poly‐T (≥ 4 T) sequence were removed from the crRNA set. The crRNA set was then screened for crRNAs that were common among H1, H3, H5, H7, and H9 influenza subtype sequences, or were at least shared among four of these subtypes.

### Design and Cloning of the crRNA Reporting System

The original expression vectors of Cas13d, crRNA, and the reporter were preserved in the laboratory. Seventeen crRNA expression plasmids expressing the crRNAs screened from the high‐throughput design were constructed using standard molecular cloning techniques and sequenced. In brief, forward and reverse oligonucleotides for each were annealed and ligated into the plasmid backbone using T4 DNA ligase (NEB; Ipswich, MA, USA). The PA, NP, and M gene segments were obtained from PR8 virus and were inserted into the reporter vector using the ClonExpress II One Step Seamless Cloning Kit (Nanjing Novizan Biotechnology Co., China). HEK293T cells were seeded at a density of 2.5×10^5^ cells per well (24‐well plate). Approximately 16 h later, cells were co‐transfected with 100 ng reporter expression plasmids, 300 ng crRNAs expression plasmids, and 600 ng Cas13d expression plasmids. Then, 48 h after co‐transfection, flow cytometry (BD FACSAria, USA) was used to detect mCherry fluorescence intensity, and quantitative real‐time PCR was performed to measure the PA, NP, and M mRNA levels. Golden gate assembly (NEB) was used to clone a tandem crRNA that expressed the three most effective crRNAs targeting PA, NP, and M, respectively.

### In Vitro Transcription and Capping of Cas13d and crRNA

The T7 promoter sequence was added to the crRNA expression vector by PCR. The mammalian codon‐optimized Cas13d expression plasmid was synthesized by VectorBuilder (VectorBuilder Inc., Guangdong, China), along with the design of the untranslated region for increasing mRNA translation efficiency. The plasmids were linearized with *BspQ* I (for Cas13d) or *Bbs* I (for crRNA) overnight at 50 °C (for *BspQ* I) or 37 °C (for *Bbs* I). Following separation by gel electrophoresis, linearized templates were recovered using a gel extraction kit (Omega, USA). In vitro transcription of Cas13d and crRNA was performed using the HiScribe T7 Quick High Yield RNA Synthesis Kit (NEB), and N1‐Me‐Pseudo UTP (Nanjing Novizan Biotechnology Co.) was added to this system. The resulting RNA was treated with DNase I (NEB) for 30 min to remove the DNA template and was then purified using the Monarch RNA Cleanup Kit (NEB). The RNA was heat‐denatured at 65 °C for 10 min before using the Vaccinia Capping System (NEB) to generate 7‐methylguanylate cap structures (Cap‐0) and mRNA Cap 2ʹ‐O‐methyltransferase (NEB) to generate the Cap‐1 structure. Cas13d mRNA was then purified using the Monarch RNA Cleanup Kit (NEB), and the concentration was measured using the Nanodrop 2000 spectrophotometer.

### Real‐Time Quantitative PCR Analysis

Total RNA was isolated from cells using an RNA Extraction Kit (Thermo Fisher Scientific, USA). RNA was reverse transcribed into first‐strand cDNA using the TransScript RT Reagent Kit (TransGen, China). Reactions were run on a LightCycler 480 (Roche, Switzerland). For relative quantification, the expression levels of mRNA for the genes were normalized to β‐actin mRNA expression levels. The primer sequences are shown in Table S3 (Supporting Information).

### Viruses and Cells

Influenza viruses A/Puerto Rico/8/1934 (A/PR/8/34, H1N1), A/California/04/2009 (Cal/04/09, H1N1), A/swine/Shandong/1207/ 2016 (Sw/SD/1207/16, H1N1),^[^ ^31^ ^]^ A/Beijing/1230/2016 (BJ/1230/16, H3N2),^[^ ^32^ ^]^ A/Anhui/1/2005 (AH/1/05, H5N1),^[^ ^33^ ^]^ A/chicken/Beijing/FH620‐L1/2017 (Ck/BJ/FH620‐L1/17, H7N9),^[^ ^33^ ^]^ and A/chicken/Hebei/LC/2008 (Ck/HB/LC/08, H9N2)^[^ ^34^ ^]^ were preserved in the laboratory. Influenza viruses were amplified in 9–11‐day‐old chicken embryos, which were specific‐pathogen‐free (Merial, Beijing, China). The human lung adenocarcinoma epithelial (A549) cells, human embryonic kidney (HEK293T) cells, primary normal human bronchial epithelial (NHBE) cells, Madin–Darby canine kidney (MDCK) cells, human immortalized keratinocytes (HaCaT) cells, human embryonic cardiac tissue‐derived (CCC‐HEH‐2) cells, and mouse embryo fibroblasts (3T3L1) were maintained in the laboratory. The cells (A549, HEK293T, NHBE, MDCK, and HaCaT) were cultivated in Dulbecco's modified Eagle's medium (DMEM) (Gibco, USA) containing 10% fetal bovine serum (Gibco), 100 µg mL^−1^ streptomycin, and 100 U mL^−1^ penicillin at 37 °C in an atmosphere containing 5% CO_2_. The CCC‐HEH‐2 cells were cultivated in DMEM containing 20% fetal bovine serum, and the 3T3L1 cells were cultivated in DMEM containing 10% newborn calf serum (Gibco). All experiments with H5 and H7 live viruses were performed in a biosafety level 3 laboratory. The TCID_50_ assay was performed in MDCK cells by inoculation with 10‐fold serially diluted viruses for 36 h, as previously reported.^[^ ^35^ ^]^ The TCID_50_ value was calculated through the Reed–Muench method.

### Phylogenetic Analyses

All available sequences of influenza viruses were downloaded from the NCBI database (www.ncbi.nlm.nih.gov). FastTree was used to construct maximum likelihood phylogenetic trees by the GTR substitution model of evolution. The trees were visualized and annotated using ChiPlot (https://www.chiplot.online/).

### Preparation and Characterization of sLNP(CRI) and LNP(CRI)

sLNP(CRI) was prepared by the thin‐film hydration method. Briefly, 7.6 mg of DOPC, 0.2 mg of DOTAP, 0.5 mg of cholesterol, 0.32 mg of 1,2‐distearoyl‐sn‐glycero‐3‐phosphoethanolamine‐N‐[methoxy(polyethylene glycol)‐2000] (DSPE‐PEG2000), and 0.8 mg of DSPE‐PEG3400‐GALA [commissioned from Ruixibio (Xi'an, China)] (GALA: WEAALAEALAEALAEHLAEALAEALEALAA) were dissolved in 4 mL of organic solvent (chloroform: methanol = 1:1, volume ratio) solution. Then, a thin lipid film was formed via rotary evaporation at 100 rpm for 20 min at 37 °C. Subsequently, 22 µg of RNA (11 µg Cas13d mRNA and 11 µg crRNA) dissolved in ultrapure water (2 mL) was added to prepare sLNP(CRI). The newly generated sLNP(CRI) was sonicated for 15 min at 200 W to produce a translucent liposome solution, which was then stored at 4 °C and sonicated for 5 min at 200 W before each experiment. The morphology of the nanoparticles was visualized using TEM (HITACHI HT7700, Japan), and the size and zeta potential was measured using a Zetasizer Nano ZS (Malvern, UK).

For the preparation of LNP(CRI), the steps were the same as those for sLNP(CRI), but DSPE‐PEG3400‐GALA was not added. For the preparation of C6‐, NR‐, and DiR‐labeled sLNP(CRI), the steps were the same as those for sLNP(CRI), but 0.5 mg of C6, NR, or DiR was added to the organic solvent, respectively.

The complexation capacity of RNA (Cas13d mRNA and crRNA) by sLNP(CRI) was evaluated by a gel retardation assay. The RNA electrophoresis buffer TBM was prepared using DEPC‐treated water (6.055 g of Tris, 3.062 g of boric acid, and 0.02 g of MgCl_2_ were dissolved in 1 L of DEPC‐treated water). Then, a 1% agarose gel in TBM was used for RNA electrophoresis. To prepare the samples, 10 µL of the sample with different N/P ratios was mixed with 2×RNA loading buffer (Beyotime, China), and then a gel retardation assay was performed on ice for 15 min at 150 V.

### Cellular Uptake Assay

A549 cells were cultured with coumarin 6 (C6)‐labeled sLNP(CRI) for 4 h at 37 °C. Then, cells were washed and suspended in PBS to detect fluorescence intensity in the FITC channel by flow cytometry (BD FACSAria).

### Lysosomal Escape Assay

A549 cells were incubated with coumarin 6 (C6)‐labeled sLNP(CRI) for 0.5, 4, 6, and 8 h at 37 °C. Cells were washed and then incubated with 50 nm Lysotracker Red DND‐99 (Invitrogen, USA) for 20 min. After washing twice with PBS, the nuclei of cells were stained by Hoechst 33324 (Beyotime) for 10 min. Subsequently, the cells were washed with PBS twice. Finally, images were captured using a confocal microscope (Leica TCS SP8).

### Stability of Tandem crRNA in Mice

The lungs of mice were collected and ground with 1 mL of PBS, and samples were subsequently centrifuged at 5000 rpm for 10 min at 4 °C. Total RNA was extracted from the supernatant using the RaPure Total RNA Kit (Magen, China). Then, crRNA was reversely transcribed into cDNA using the PrimeScript RT Reagent Kit (Takara, Japan) (Reverse primer: ATTTCTTCGGAGACAATGCA). The reverse‐transcription mixture was amplified by real‐time quantitative PCR analysis using the SYBR Green qPCR mixture (Takara, Japan) (Forward: 5′‐CTGCAGTCCTCGCTCACTC‐3′; Reverse: 5′‐GGAGACAATGCAGAGT‐3′).

### Detection of Sialic acid Receptor Expression Levels

A549, HaCaT, CCC‐HEH‐2, and 3T3L1 cells were fixed with 4% paraformaldehyde for 15 min followed by permeabilization with 0.1% Triton X‐100 for 15 min. Then, cells were incubated with 10 g ml^−1^ biotinylated lectins [*Sambucus sieboldiana agglutinin* (Vectorlabs, USA) and *Maackia amurensis agglutinin* (Vectorlabs)] at 4 °C overnight. Cells were washed with PBS and then incubated with 10 g ml^−1^ DyLight 549‐labeled streptavidin (Vectorlabs) for 8 h. After washing with PBS, cells were stained with DAPI (Beyotime) for 7 min, then washed again with PBS.

### 3D Spheroid Penetration Assay

The bottom agar‐medium mixture (DMEM, 0.8% agarose) was added to the wells of 96‐well plates and allowed to solidify. Plates were exposed under ultraviolet light for 30 min. Subsequently, cells were seeded at a density of 2000 cells per well in 100 µL of medium. C6‐labeled sLNP(CRI) at a C6 concentration of 1.5 µg mL^−1^ were added to the 3D spheroids when the spheroid diameter was 200–300 µm. After incubation for 4 h, confocal microscopy (Leica TCS SP8) was used to confirm the exact location of C6‐sLNP(CRI) in the spheroids by scanning from top to bottom at a 100 µm depth.

### In Vivo Localization and Biodistribution

Mice were administered DiR‐labeled sLNP(CRI) or DiR‐labeled LNP(CRI) via the tail vein. Then, their hearts, livers, spleens, lungs, kidneys, brains, and intestines were harvested at various time points after injection. Each mouse was injected with the same dosage of DiR (2 mg kg^˗1^) and the bioluminescent data were analyzed by IVIS (IVIS Lumina Series III, PerkinElmer, USA).

### Animal Experiments

BALB/c female mice (6–8 weeks old) were maintained under specific‐pathogen‐free conditions. Mice were anesthetized with zoletil (20 µg g^˗1^) and intranasally inoculated with 100 TCID_50_ of PR8 virus diluted in 50 µL PBS; six mice from each group were euthanized, and viral titers in their lungs were determined using a TCID_50_ assay. Daily monitoring of body weight and survival rates was conducted from day 0 to day 14. When weight loss reached more than 25% of the initial body weight, mice were euthanized. Mouse inflammatory cytokines in the lungs were measured using the cytometric bead array system (BD).

### Statistical Analysis

All values are shown as the mean ± SD. At least three independent biological repeats were performed for all experiments. A two‐tailed Student's *t*‐test was used to compare the differences between the two groups. All statistical analyses were performed using GraphPad Prism software v.7.00 (GraphPad Software Inc., San Diego, CA, USA). *P*‐values less than 0.05 were considered statistically significant.

### Ethical Statement

This research was conducted in compliance with the guidelines stipulated in the Guide for the Care and Use of Laboratory Animals issued by the Ministry of Science and Technology of the People's Republic of China. The protocols involving animals in this study received official approval from the Committee on the Ethics of Laboratory Animals of China Agricultural University (approval number: AW11203202‐2‐2).

## Conflict of Interest

The authors declare no conflict of interest.

## Author Contributions

ZL.W., C.Z., R.Z., X.H., J.L., X.M., and Y.S. designed and performed the experiments. H.A., Z.W., and M.C. analyzed the data. Y.L., Q.T., L.L., H.S., and J.P. provided the facilities. ZL.W., X.M., and Y.S. wrote the manuscript. X.M., and Y.S. provided financial support.

## Supporting information



Supporting Information

Supporting Data Files

## Data Availability

The data that support the findings of this study are available from the corresponding author upon reasonable request.
