# Supplementary material for: A Susceptible Cell‐Selective Delivery (SCSD) of mRNA‐Encoded Cas13d Against Influenza Infection
Source: Adv Sci (Weinh). 2025 Jan 10;12(9):2414651. doi: 10.1002/advs.202414651 (PMC11884569; doi:10.1002/advs.202414651)
Supplement: Supplementary file 1 — Supporting Information [file ADVS-12-2414651-s002.docx]

Supporting Information

A Susceptible Cell-Selective Delivery (SCSD) of mRNA-Encoded Cas13d Against Influenza Infection

*Zhuanli Wu, Chengcheng Zhao, Hui Ai, Zhen Wang, Mingyue Chen, Yanli Lyu, Qi Tong, Litao Liu, Honglei Sun, Juan Pu, Ran Zhang, Xiaoxiang Hu, Jinhua Liu,* *Xiaowei Ma, * and Yipeng Sun**

***Correspondence:** Xiaowei Ma, Yipeng Sun

Table S1. Number of influenza PA/NP/M segments analyzed in each subtype.

| Virus subtype | Number of sequences analyzed | |
| --- | --- | --- |
| H1 | M | 9269 |
|  | NP | 9140 |
|  | PA | 8821 |
| H3 | M | 17801 |
|  | NP | 17056 |
|  | PA | 16549 |
| H5 | M | 1072 |
|  | NP | 1068 |
|  | PA | 1048 |
| H7 | M | 285 |
|  | NP | 288 |
|  | PA | 277 |
| H9 | M | 1225 |
|  | NP | 1199 |
|  | PA | 1196 |

Table S2. Spacer sequences of crRNAs in the 17 synthesized crRNA expression vectors.

| PA1-crRNA | GAACGGCTGCATTGAGGGCAA |
| --- | --- |
| PA2-crRNA | CCGAACGGCTGCATTGAGGGCA |
| PA3-crRNA | CGAACGGCTGCATTGAGGGCAA |
| PA4-crRNA | CGAACGGCTGCATTGAGGGCA |
| PA5-crRNA | CCGAACGGCTGCATTGAGGGC |
| NP1-crRNA | CTTATTTCTTCGGAGACAATGCAGA |
| NP2-crRNA | AAAGGCAACGAACCCGATCGT |
| NP3-crRNA | TCTTATTTCTTCGGAGACAATGC |
| NP4-crRNA | GATCTTATTTCTTCGGAGACAATGC |
| NP5-crRNA | AAAGGCAACGAACCCGATCGTGCCTTC |
| NP6-crRNA | GCAACGAACCCGATCGTGCCTTC |
| M1 -crRNA | ACGCTCACCGTGCCCAGTGAG |
| M2-crRNA | AGTGAGCGAGGACTGCAGCGTAGAC |
| M3-crRNA | AGGCCCCCTCAAAGCCGAGAT |
| M4-crRNA | GCCCAGTGAGCGAGGACTGCAGCGTAG |
| M5-crRNA | CAGTGAGCGAGGACTGCAGCGTA |
| M6-crRNA | CGCTCACCGTGCCCAGTGAGCGAG |

Table S3. The sequences of primers used for RT-qPCR in the study.

| **Gene** | **Forward** | **Reverse** |
| --- | --- | --- |
| **Influenza virus Gene** | | |
| **PA** | GAACCAGGGAAGGAAGACGG | ACTTGACCTATTGCCGTCCG |
| **NP** | CTGCGGTGAAAGGAGTTGGA | AAGCAACCCTTGTCCTTCGT |
| **M** | ACCAGAAGCGAATGGGAGTG | TCAGGCACTCCTTCCGTAGA |
| **Human Gene** | | |
| **β-actin** | CACCATTGGCAATGAGCGGTTC | AGGTCTTTGCGGATGTCCACG |
| **IL-6** | AGACAGCCACTCACCTCTTCAG | TTCTGCCAGTGCCTCTTTGCTG |
| **CCL2** | AGAATCACCAGCAGCAAGTGTCC | TCCTGAACCCACTTCTGCTTGG |
| **IFN-β** | GCACAACAGGTAGTAGGCGA | TGGAAAGAGCTGTCGTGGAG |
| **Mouse Gene** | | |
| **β-actin** | CATTGCTGACAGGATGCAGAAGG | TGCTGGAAGGTGGACAGTGAGG |
| **IL-6** | TACCACTTCACAAGTCGGAGGC | CTGCAAGTGCATCATCGTTGTTC |
| **CCL2** | GCTACAAGAGGATCACCAGCAG | GTCTGGACCCATTCCTTCTTGG |
| **IFN-β** | ATGAGTGGTGGTTGCAGGC | TGACCTTTCAAATGCAGTAGATTCA |


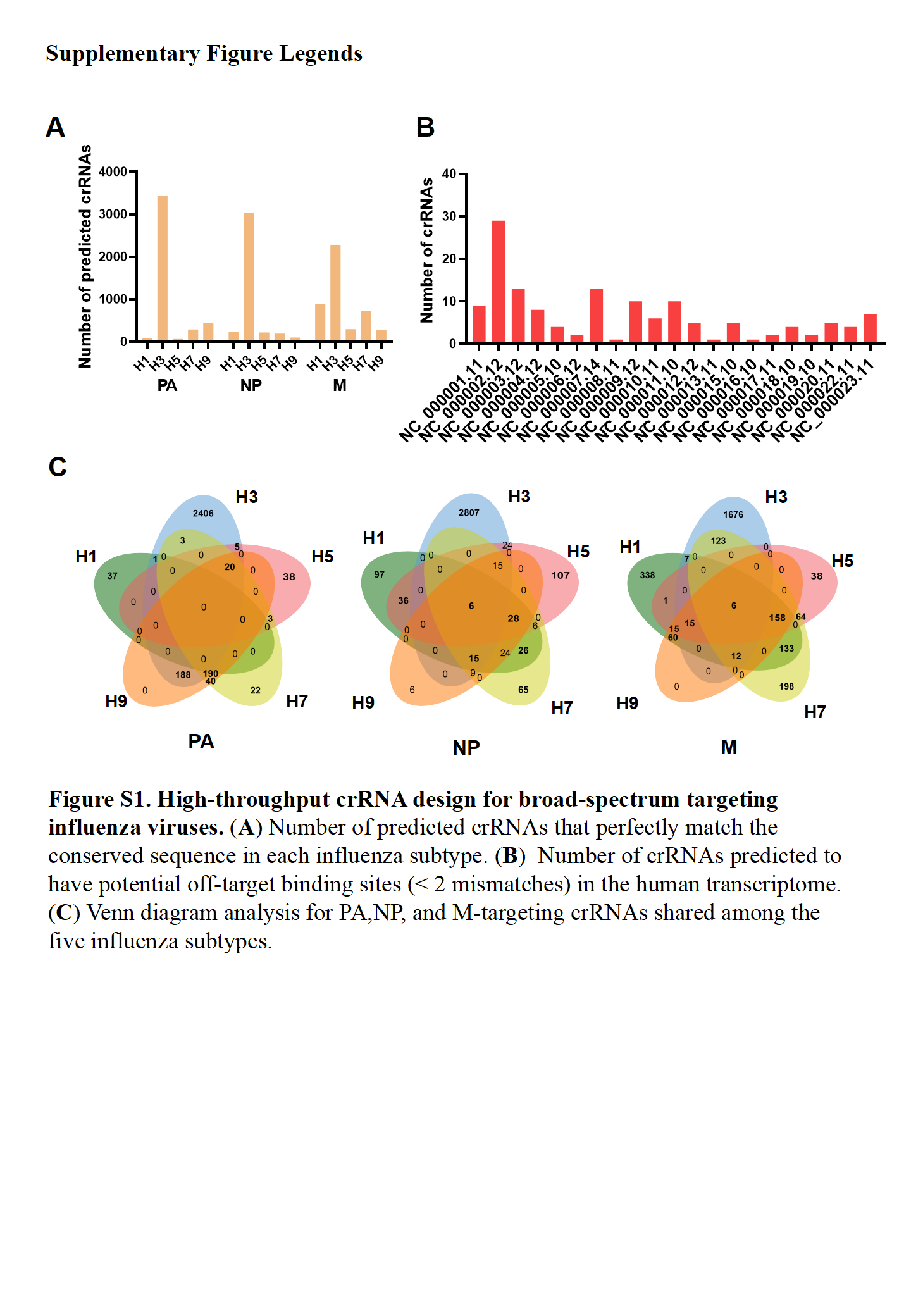


**Figure S1. High-throughput crRNA design for broad-spectrum targeting influenza viruses. A**, Number of predicted crRNAs that perfectly match the conserved sequence in each influenza subtype. **B**, Number of crRNAs predicted to have potential off-target binding sites (≤ 2 mismatches) in the human transcriptome. **C**, Venn diagram analysis for PA,NP, and M-targeting crRNAs shared among the five influenza subtypes.


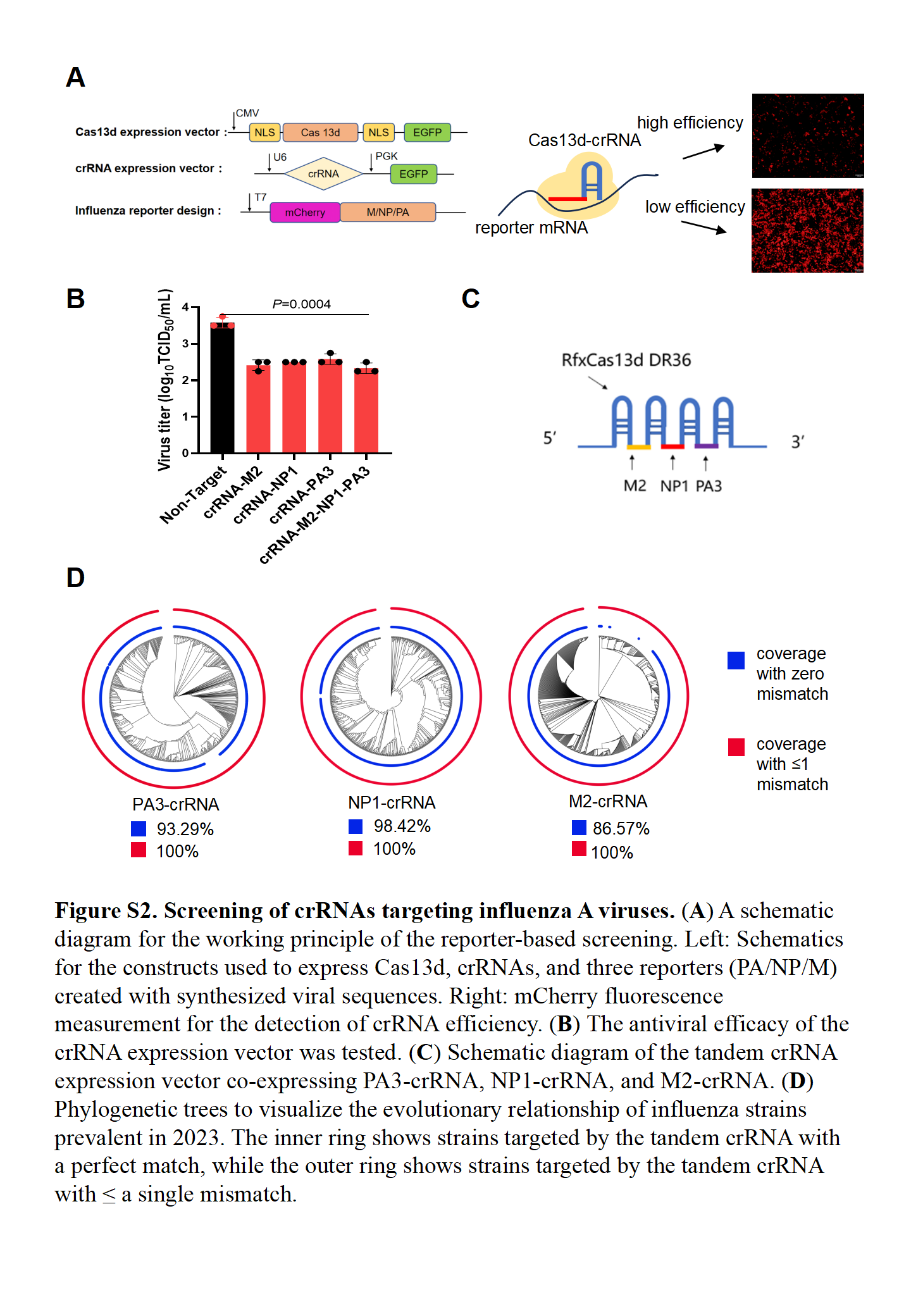


**Figure S2. Screening of crRNAs targeting influenza A viruses.** **A,** A schematic diagram for the working principle of the reporter-based screening. Left: Schematics for the constructs used to express Cas13d, crRNAs, and three reporters (PA/NP/M) created with synthesized viral sequences. Right: mCherry fluorescence measurement for the detection of crRNA efficiency. **B,** The antiviral efficacy of the crRNA expression vector was tested. **C,** Schematic diagram of the tandem crRNA expression vector co-expressing PA3-crRNA, NP1-crRNA, and M2-crRNA. **D,** Phylogenetic trees to visualize the evolutionary relationship of influenza strains prevalent in 2023. The inner ring shows strains targeted by the tandem crRNA with a perfect match, while the outer ring shows strains targeted by the tandem crRNA with ≤ a single mismatch.


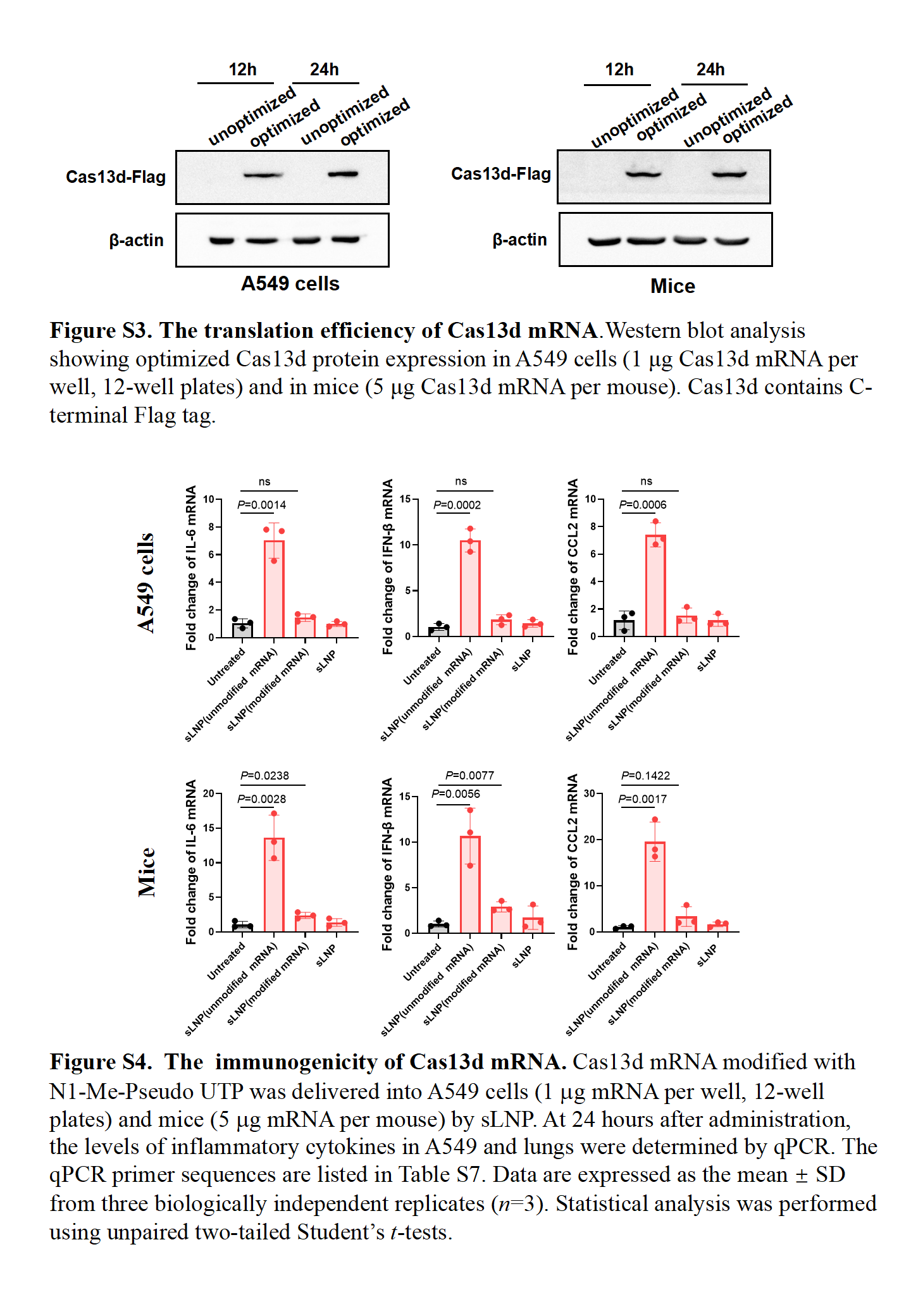


**Figure S3. The translation efficiency of Cas13d mRNA.** Western blot analysis showing optimized Cas13d protein expression in A549 cells (1 μg Cas13d mRNA per well, 12-well plates) and in mice (5 μg Cas13d mRNA per mouse). Cas13d contains C-terminal Flag tag.


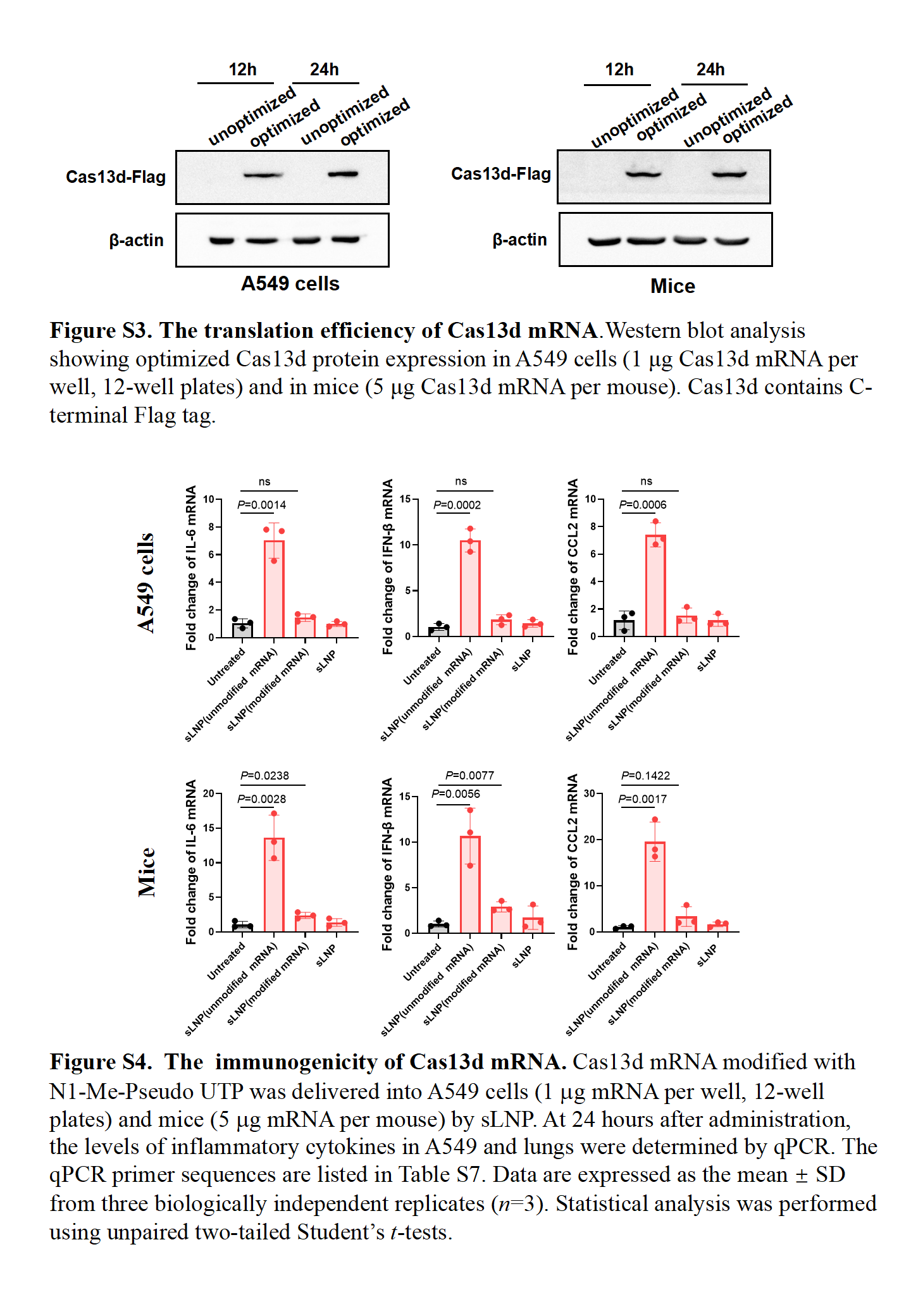


**Figure S4. The immunogenicity of Cas13d mRNA.** Cas13d mRNA modified with N1-Me-Pseudo UTP was delivered into A549 cells (1 μg mRNA per well, 12-well plates) and mice (5 μg mRNA per mouse) by sLNP. At 24 hours after administration, the levels of inflammatory cytokines in A549 and lungs were determined by qPCR. The qPCR primer sequences are listed in Table S7. Data are expressed as the mean ± SD from three biologically independent replicates (*n*=3). Statistical analysis was performed using unpaired two-tailed Student’s *t*-tests.


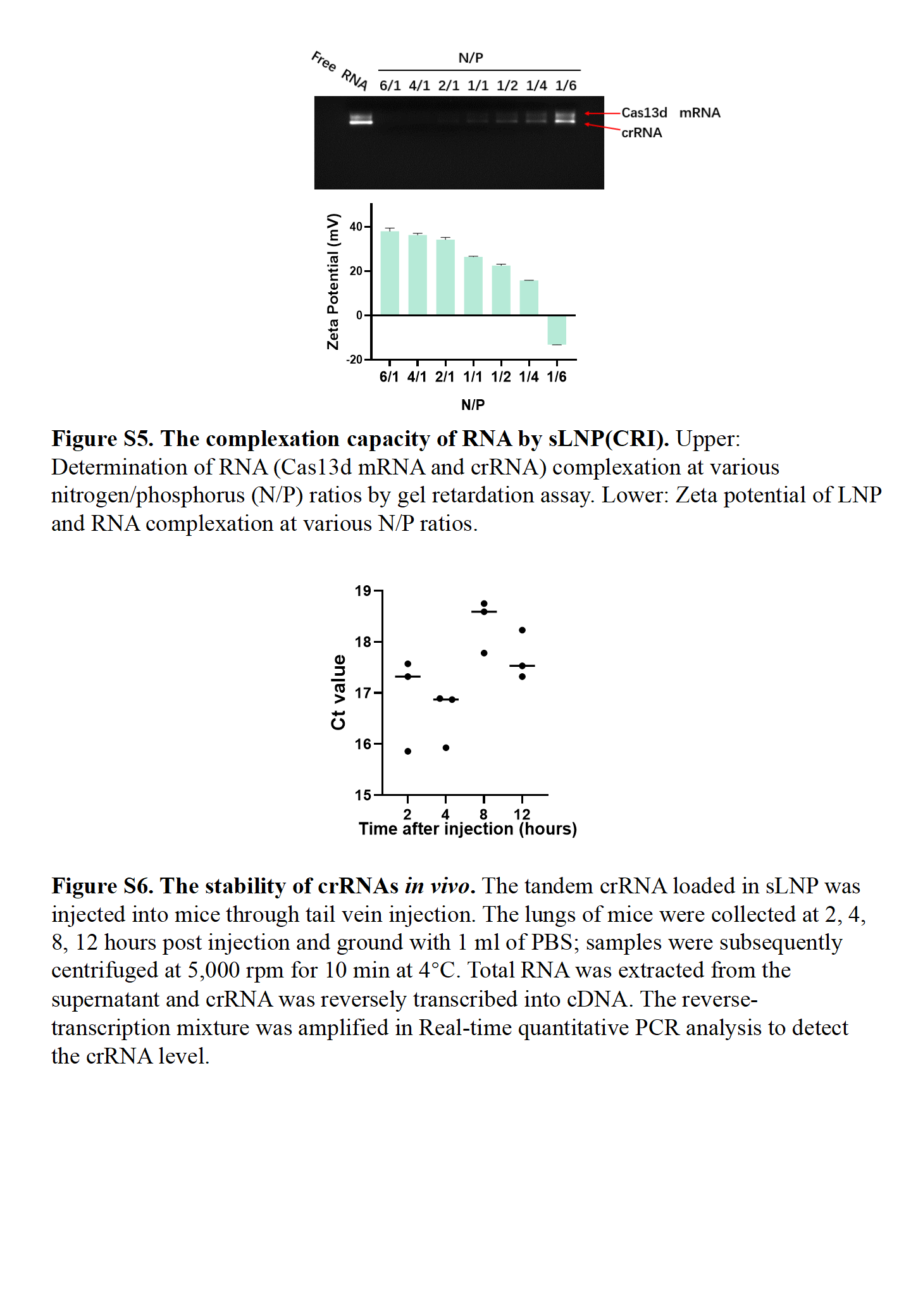


**Figure S5. The complexation capacity of RNA by sLNP(CRI).** Upper: Determination of RNA (Cas13d mRNA and crRNA) complexation at various nitrogen/phosphorus (N/P) ratios by gel retardation assay. Lower: Zeta potential of LNP and RNA complexation at various N/P ratios.


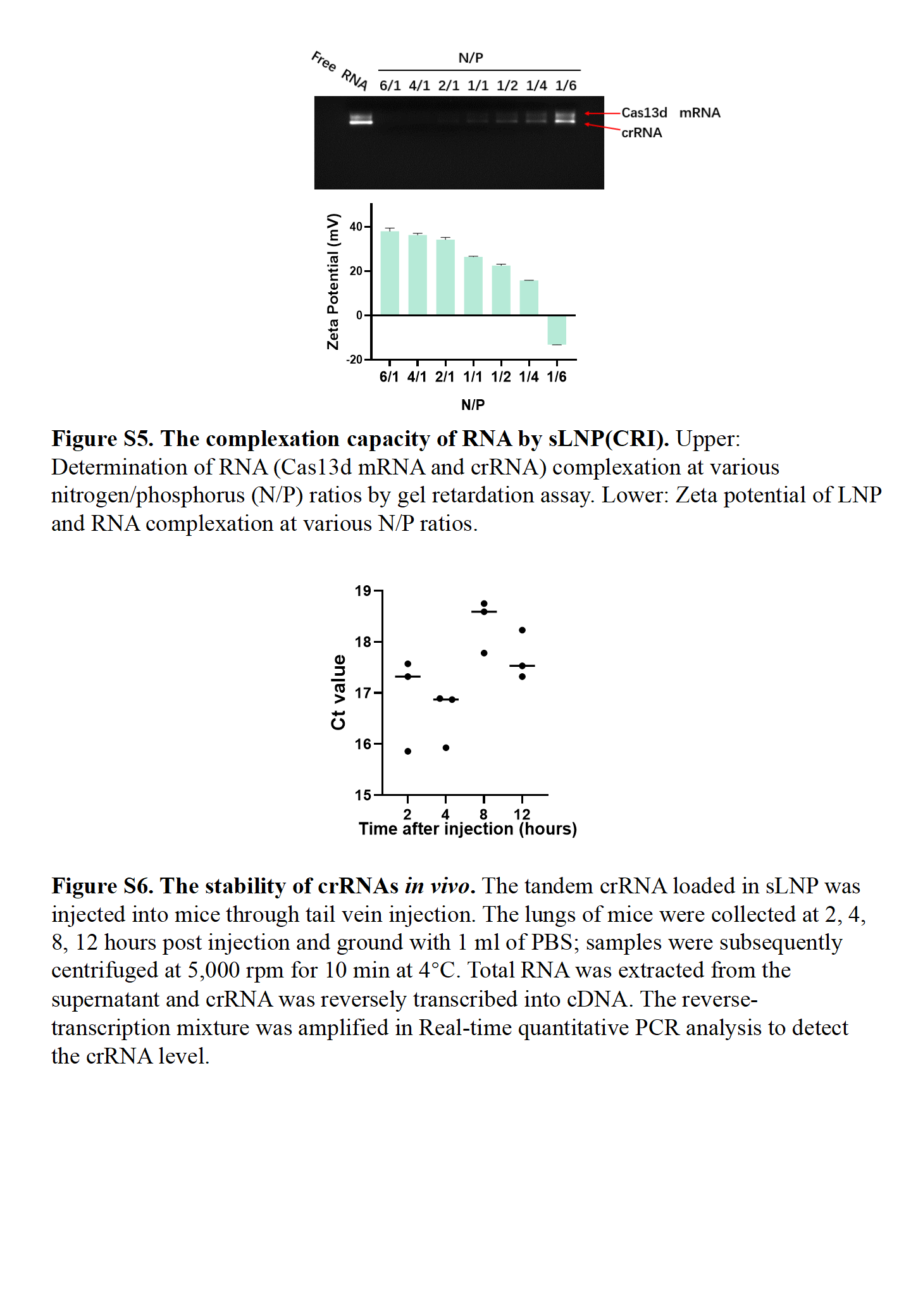


**Figure S6. The stability of crRNAs *in vivo*.** The tandem crRNA loaded in sLNP was injected into mice through tail vein injection. The lungs of mice were collected at 2, 4, 8, 12 hours post injection and ground with 1 mL of PBS; samples were subsequently centrifuged at 5,000 rpm for 10 min at 4 °C. Total RNA was extracted from the supernatant and crRNA was reversely transcribed into cDNA. The reverse-transcription mixture was amplified in Real-time quantitative PCR analysis to detect the crRNA level.


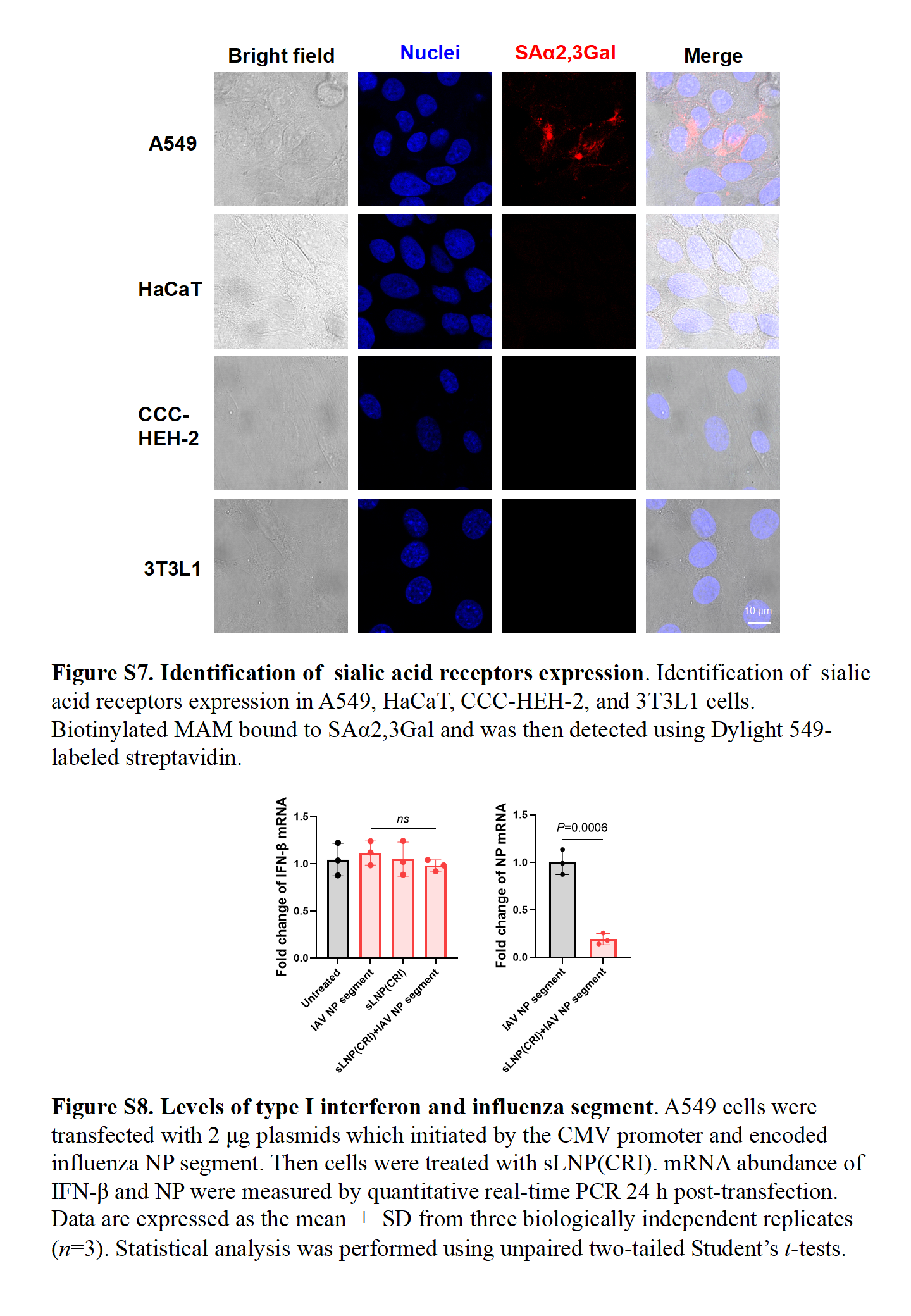


**Figure S7. Identification of sialic acid receptors expression.** Identification of sialic acid receptors expression in A549, HaCaT, CCC-HEH-2, and 3T3L1 cells. Biotinylated MAM bound to SAα2,3Gal and was then detected using Dylight 549-labeled streptavidin.


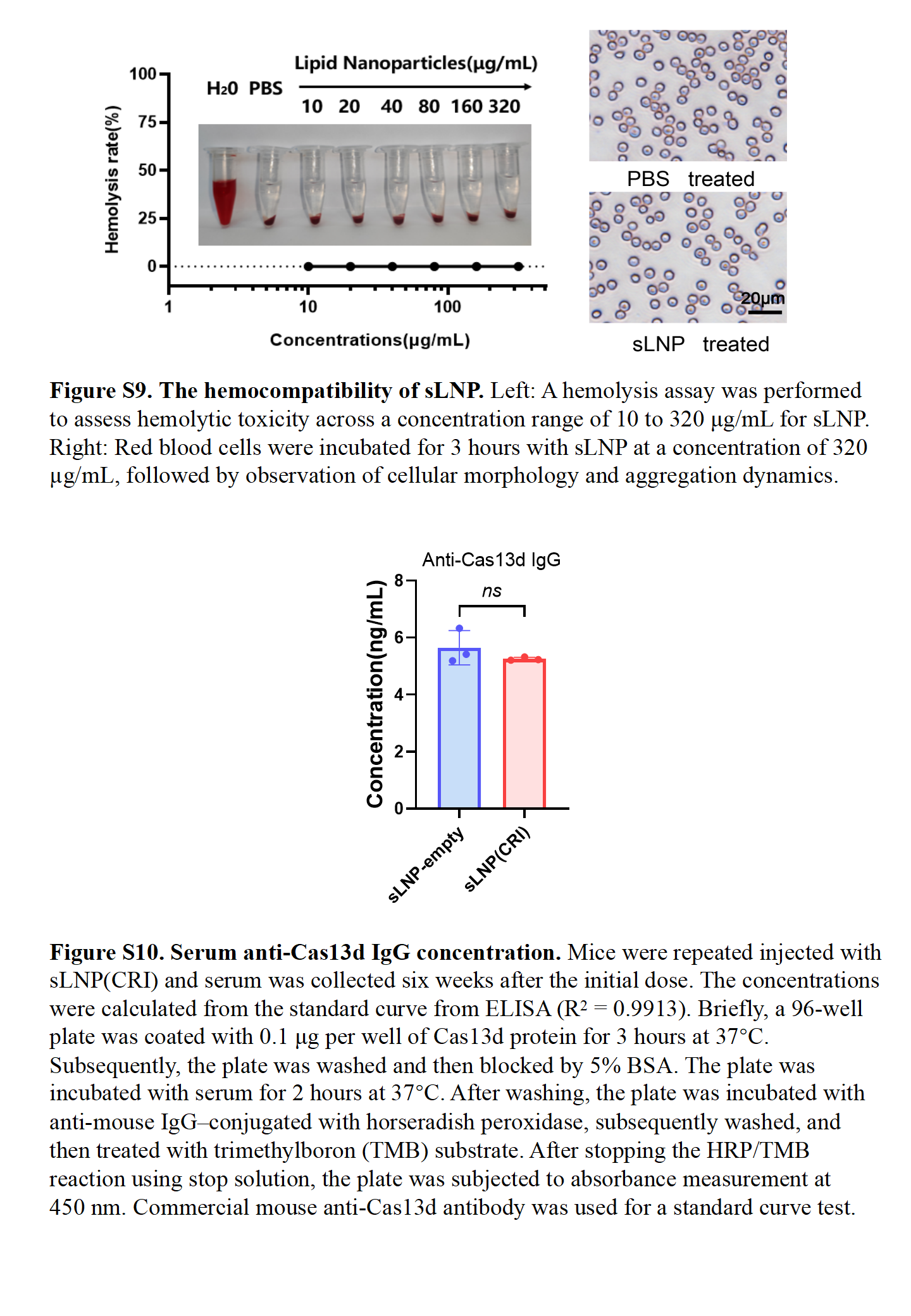


**Figure S8. The hemocompatibility of sLNP.** Left: A hemolysis assay was performed to assess hemolytic toxicity across a concentration range of 10 to 320 μg/mL for sLNP. Right: Red blood cells were incubated for 3 hours with sLNP at a concentration of 320 µg/mL, followed by observation of cellular morphology and aggregation dynamics.


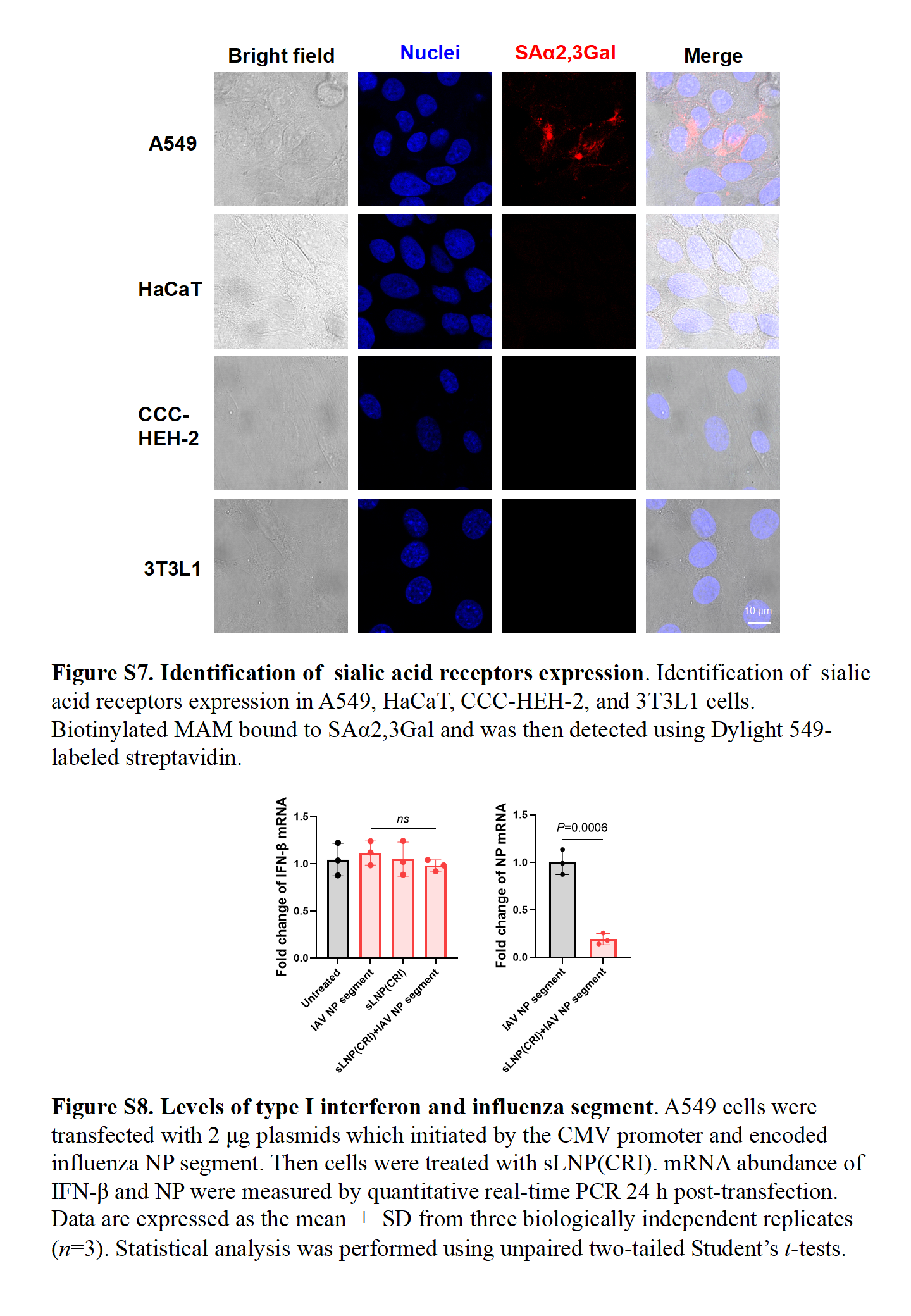


**Figure S9. Levels of type I interferon and influenza segment.** A549 cells were transfected with 2 μg plasmids which initiated by the CMV promoter and encoded influenza NP segment. Then cells were treated with sLNP(CRI). mRNA abundance of IFN-β and NP were measured by quantitative real-time PCR 24 h post-transfection. Data are expressed as the mean ± SD from three biologically independent replicates (*n*=3). Statistical analysis was performed using unpaired two-tailed Student’s *t*-tests.


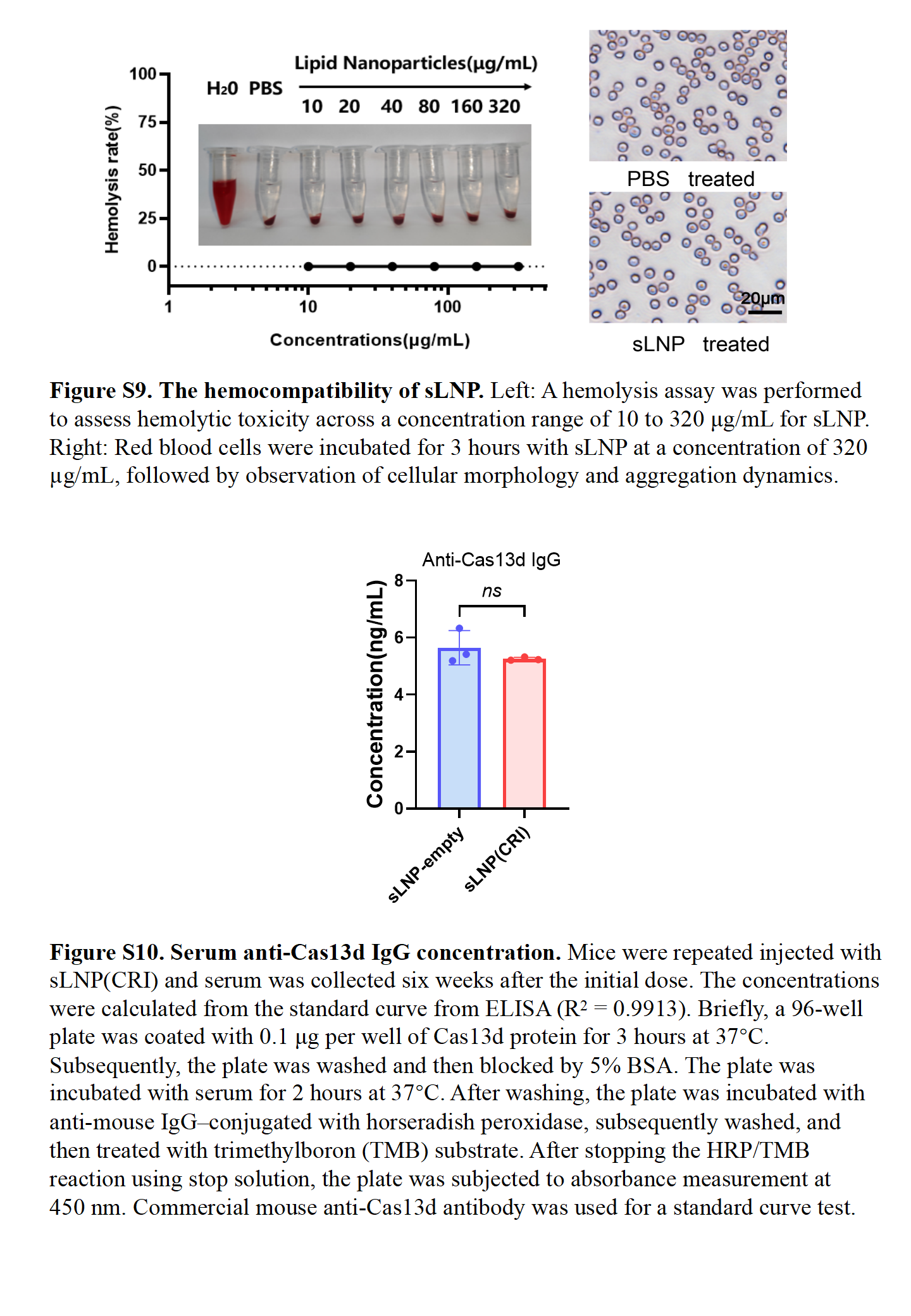


**Figure S10. Serum anti-Cas13d IgG concentration.** Mice were repeated injected with sLNP(CRI) and serum was collected six weeks after the initial dose. The concentrations were calculated from the standard curve from ELISA (R^2^ = 0.9913). Briefly, a 96-well plate was coated with 0.1 μg per well of Cas13d protein for 3 hours at 37 °C. Subsequently, the plate was washed and then blocked by 5% BSA. The plate was incubated with serum for 2 hours at 37 °C. After washing, the plate was incubated with anti-mouse IgG–conjugated with horseradish peroxidase, subsequently washed, and then treated with trimethylboron (TMB) substrate. After stopping the HRP/TMB reaction using stop solution, the plate was subjected to absorbance measurement at 450 nm. Commercial mouse anti-Cas13d antibody was used for a standard curve test.


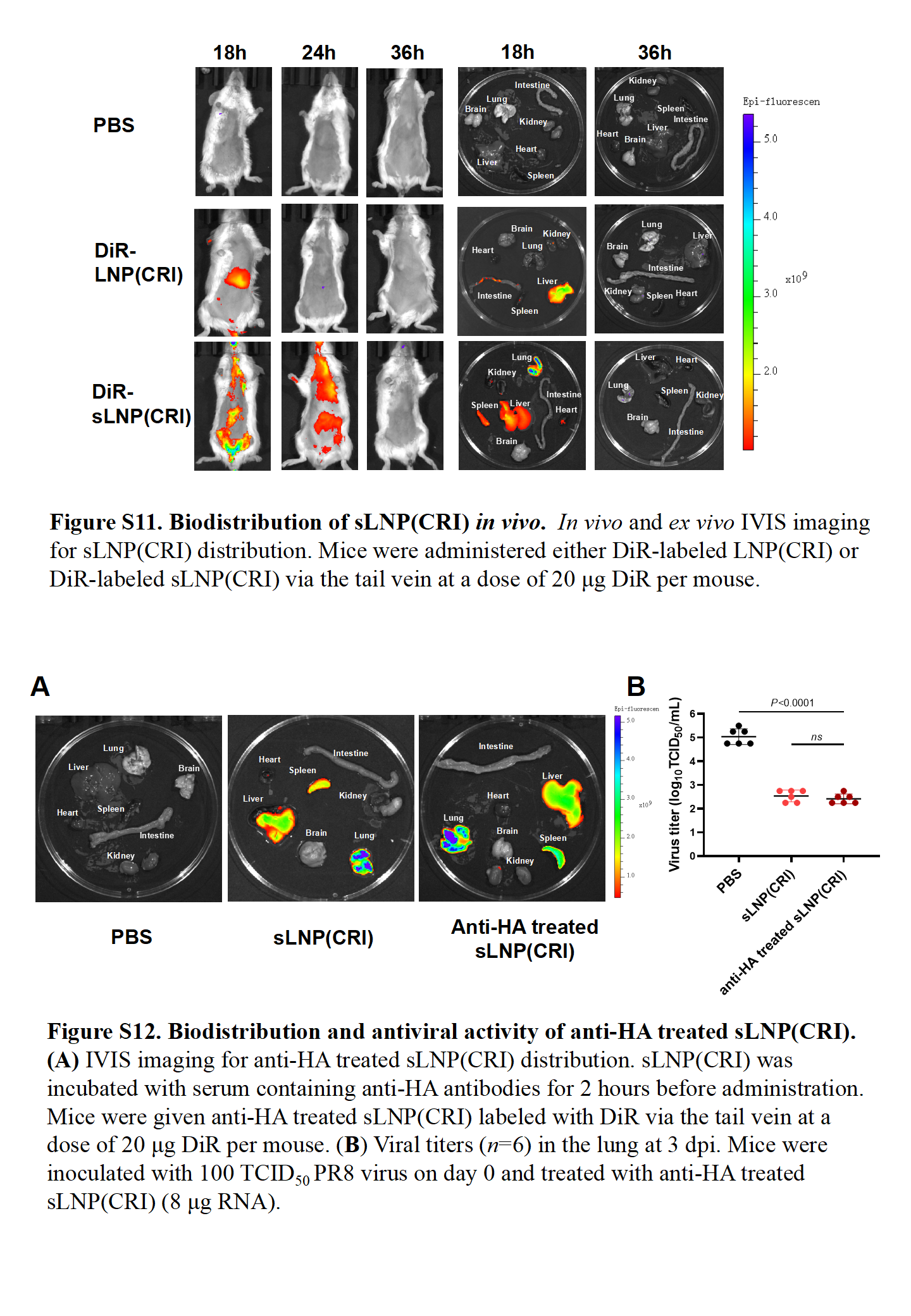


**Figure S11. Biodistribution of sLNP(CRI) *in vivo*.** *In vivo* and *ex vivo* IVIS imaging for sLNP(CRI) distribution. Mice were administered either DiR-labeled LNP(CRI) or DiR-labeled sLNP(CRI) via the tail vein at a dose of 20 μg DiR per mouse.


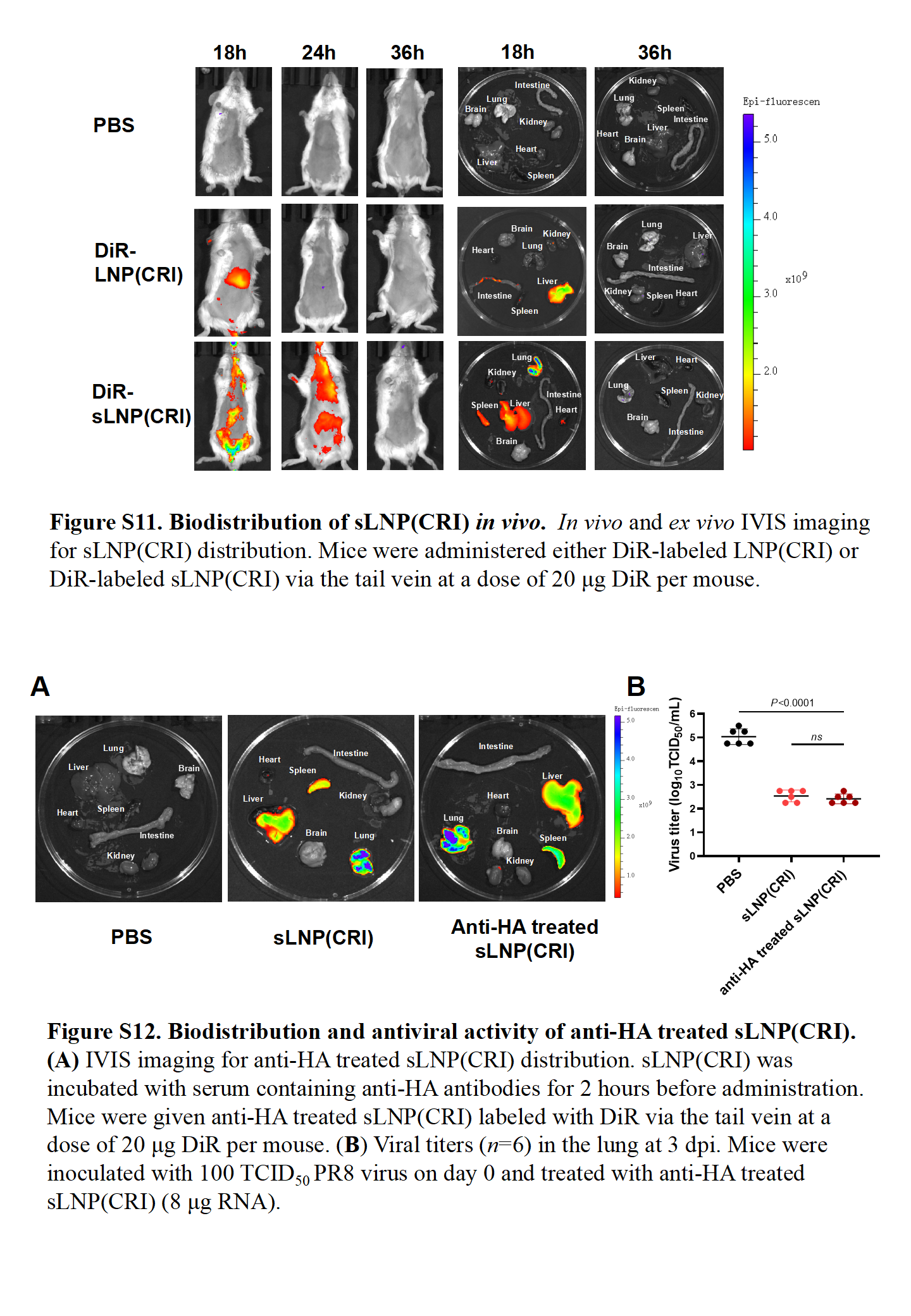


**Figure S12. Biodistribution and antiviral activity of anti-HA treated sLNP(CRI).** **A,** IVIS imaging for anti-HA treated sLNP(CRI) distribution. sLNP(CRI) was incubated with serum containing anti-HA antibodies for 2 hours before administration. Mice were given anti-HA treated sLNP(CRI) labeled with DiR via the tail vein at a dose of 20 μg DiR per mouse. **B,** Viral titers (*n*=6) in the lung at 3 dpi. Mice were inoculated with 100 TCID_50_ PR8 virus on day 0 and treated with anti-HA treated sLNP(CRI) (8 μg RNA).
