# Supplementary material for: A Susceptible Cell‐Selective Delivery (SCSD) of mRNA‐Encoded Cas13d Against Influenza Infection
Source: Adv Sci (Weinh). 2025 Jan 10;12(9):2414651. doi: 10.1002/advs.202414651 (PMC11884569; doi:10.1002/advs.202414651)
Supplement: Supplementary file 2 — Supporting Data Files [file ADVS-12-2414651-s001.zip › PD-Data File 5.docx]

**Cas13d sequence without optimization:**

ATGCCTAAAAAGAAAAGAAAGGTGATCGAAAAAAAAAAGTCCTTCGCCAAGGGCATGGGCGTGAAGTCCACACTCGTGTCCGGCTCCAAAGTGTACATGACAACCTTCGCCGAAGGCAGCGACGCCAGGCTGGAAAAGATCGTGGAGGGCGACAGCATCAGGAGCGTGAATGAGGGCGAGGCCTTCAGCGCTGAAATGGCCGATAAAAACGCCGGCTATAAGATCGGCAACGCCAAATTCAGCCATCCTAAGGGCTACGCCGTGGTGGCTAACAACCCTCTGTATACAGGACCCGTCCAGCAGGATATGCTCGGCCTGAAGGAAACTCTGGAAAAGAGGTACTTCGGCGAGAGCGCTGATGGCAATGACAATATTTGTATCCAGGTGATCCATAACATCCTGGACATTGAAAAAATCCTCGCCGAATACATTACCAACGCCGCCTACGCCGTCAACAATATCTCCGGCCTGGATAAGGACATTATTGGATTCGGCAAGTTCTCCACAGTGTATACCTACGACGAATTCAAAGACCCCGAGCACCATAGGGCCGCTTTCAACAATAACGATAAGCTCATCAACGCCATCAAGGCCCAGTATGACGAGTTCGACAACTTCCTCGATAACCCCAGACTCGGCTATTTCGGCCAGGCCTTTTTCAGCAAGGAGGGCAGAAATTACATCATCAATTACGGCAACGAATGCTATGACATTCTGGCCCTCCTGAGCGGACTGAGGCACTGGGTGGTCCATAACAACGAAGAAGAGTCCAGGATCTCCAGGACCTGGCTCTACAACCTCGATAAGAACCTCGACAACGAATACATCTCCACCCTCAACTACCTCTACGACAGGATCACCAATGAGCTGACCAACTCCTTCTCCAAGAACTCCGCCGCCAACGTGAACTATATTGCCGAAACTCTGGGAATCAACCCTGCCGAATTCGCCGAACAATATTTCAGATTCAGCATTATGAAAGAGCAGAAAAACCTCGGATTCAATATCACCAAGCTCAGGGAAGTGATGCTGGACAGGAAGGATATGTCCGAGATCAGGAAAAATCATAAGGTGTTCGACTCCATCAGGACCAAGGTCTACACCATGATGGACTTTGTGATTTATAGGTATTACATCGAAGAGGATGCCAAGGTGGCTGCCGCCAATAAGTCCCTCCCCGATAATGAGAAGTCCCTGAGCGAGAAGGATATCTTTGTGATTAACCTGAGGGGCTCCTTCAACGACGACCAGAAGGATGCCCTCTACTACGATGAAGCTAATAGAATTTGGAGAAAGCTCGAAAATATCATGCACAACATCAAGGAATTTAGGGGAAACAAGACAAGAGAGTATAAGAAGAAGGACGCCCCTAGACTGCCCAGAATCCTGCCCGCTGGCCGTGATGTTTCCGCCTTCAGCAAACTCATGTATGCCCTGACCATGTTCCTGGATGGCAAGGAGATCAACGACCTCCTGACCACCCTGATTAATAAATTCGATAACATCCAGAGCTTCCTGAAGGTGATGCCTCTCATCGGAGTCAACGCTAAGTTCGTGGAGGAATACGCCTTTTTCAAAGACTCCGCCAAGATCGCCGATGAGCTGAGGCTGATCAAGTCCTTCGCTAGAATGGGAGAACCTATTGCCGATGCCAGGAGGGCCATGTATATCGACGCCATCCGTATTTTAGGAACCAACCTGTCCTATGATGAGCTCAAGGCCCTCGCCGACACCTTTTCCCTGGACGAGAACGGAAACAAGCTCAAGAAAGGCAAGCACGGCATGAGAAATTTCATTATTAATAACGTGATCAGCAATAAAAGGTTCCACTACCTGATCAGATACGGTGATCCTGCCCACCTCCATGAGATCGCCAAAAACGAGGCCGTGGTGAAGTTCGTGCTCGGCAGGATCGCTGACATCCAGAAAAAACAGGGCCAGAACGGCAAGAACCAGATCGACAGGTACTACGAAACTTGTATCGGAAAGGATAAGGGCAAGAGCGTGAGCGAAAAGGTGGACGCTCTCACAAAGATCATCACCGGAATGAACTACGACCAATTCGACAAGAAAAGGAGCGTCATTGAGGACACCGGCAGGGAAAACGCCGAGAGGGAGAAGTTTAAAAAGATCATCAGCCTGTACCTCACCGTGATCTACCACATCCTCAAGAATATTGTCAATATCAACGCCAGGTACGTCATCGGATTCCATTGCGTCGAGCGTGATGCTCAACTGTACAAGGAGAAAGGCTACGACATCAATCTCAAGAAACTGGAAGAGAAGGGATTCAGCTCCGTCACCAAGCTCTGCGCTGGCATTGATGAAACTGCCCCCGATAAGAGAAAGGACGTGGAAAAGGAGATGGCTGAAAGAGCCAAGGAGAGCATTGACAGCCTCGAGAGCGCCAACCCCAAGCTGTATGCCAATTACATCAAATACAGCGACGAGAAGAAAGCCGAGGAGTTCACCAGGCAGATTAACAGGGAGAAGGCCAAAACCGCCCTGAACGCCTACCTGAGGAACACCAAGTGGAATGTGATCATCAGGGAGGACCTCCTGAGAATTGACAACAAGACATGTACCCTGTTCAGAAACAAGGCCGTCCACCTGGAAGTGGCCAGGTATGTCCACGCCTATATCAACGACATTGCCGAGGTCAATTCCTACTTCCAACTGTACCATTACATCATGCAGAGAATTATCATGAATGAGAGGTACGAGAAAAGCAGCGGAAAGGTGTCCGAGTACTTCGACGCTGTGAATGACGAGAAGAAGTACAACGATAGGCTCCTGAAACTGCTGTGTGTGCCTTTCGGCTACTGTATCCCCAGGTTTAAGAACCTGAGCATCGAGGCCCTGTTCGATAGGAACGAGGCCGCCAAGTTCGACAAGGAGAAAAAGAAGGTGTCCGGCAATTCCCCCAAGAAGAAGAGGAAAGTCGACTACAAGGACGACGATGACAAGTAA

**Codon-optimized and modified Cas13d sequence:**

AATAAACTAGTATTCTTCTGGTCCCCACAGACTCAGAGAGAACCCGCCACCATGCCTAAAAAGAAAAGAAAGGTGGGTTCTGGTATCGAGAAGAAGAAAAGCTTCGCCAAGGGCATGGGAGTGAAAAGCACCCTGGTGTCCGGCTCTAAGGTGTACATGACCACATTTGCTGAGGGAAGCGACGCCAGGCTGGAGAAGATCGTGGAGGGCGATAGCATCAGATCCGTGAACGAGGGAGAGGCTTTCAGCGCCGAGATGGCTGACAAGAACGCTGGCTACAAGATCGGAAACGCCAAGTTTTCCCACCCAAAGGGCTACGCCGTGGTGGCTAACAACCCACTGTACACCGGACCAGTGCAGCAGGACATGCTGGGACTGAAGGAGACACTGGAGAAGAGGTACTTCGGCGAGTCCGCCGACGGAAACGATAACATCTGCATCCAGGTCATCCACAACATCCTGGATATCGAGAAGATCCTGGCTGAGTACATCACAAACGCCGCTTACGCCGTGAACAACATCTCCGGCCTGGACAAGGATATCATCGGCTTCGGAAAGTTTTCTACCGTGTACACATACGACGAGTTCAAGGATCCAGAGCACCACCGGGCCGCTTTTAACAACAACGACAAGCTGATCAACGCCATCAAGGCTCAGTACGACGAGTTCGATAACTTTCTGGATAACCCCAGGCTGGGCTACTTCGGACAGGCTTTCTTTTCTAAGGAGGGCAGAAACTACATCATCAACTACGGAAACGAGTGTTACGACATCCTGGCCCTGCTGAGCGGACTGAGGCACTGGGTGGTGCACAACAACGAGGAGGAGTCTCGGATCAGCCGCACCTGGCTGTACAACCTGGACAAGAACCTGGATAACGAGTACATCTCCACACTGAACTACCTGTACGACAGGATCACCAACGAGCTGACAAACAGCTTCTCCAAGAACTCTGCCGCTAACGTGAACTACATCGCTGAGACCCTGGGCATCAACCCAGCTGAGTTCGCTGAGCAGTACTTCAGATTTTCCATCATGAAGGAGCAGAAGAACCTGGGCTTCAACATCACAAAGCTGAGAGAAGTGATGCTGGACAGAAAGGATATGTCCGAGATCAGGAAGAACCACAAGGTGTTCGATTCTATCAGAACCAAGGTGTACACAATGATGGACTTTGTGATCTACAGGTACTACATCGAGGAGGATGCCAAGGTGGCCGCTGCCAACAAGAGCCTGCCCGACAACGAGAAGTCTCTGAGCGAGAAGGATATCTTCGTGATCAACCTGAGAGGCTCCTTTAACGACGATCAGAAGGACGCTCTGTACTACGATGAGGCCAACAGGATCTGGAGAAAGCTGGAGAACATCATGCACAACATCAAGGAGTTCCGGGGAAACAAGACCCGCGAGTACAAGAAGAAGGACGCTCCAAGGCTGCCTAGGATCCTGCCTGCTGGAAGGGACGTGAGCGCCTTCAGCAAGCTGATGTACGCCCTGACAATGTTTCTGGACGGAAAGGAGATCAACGATCTGCTGACCACACTGATCAACAAGTTCGACAACATCCAGTCTTTTCTGAAAGTGATGCCTCTGATCGGCGTGAACGCTAAGTTCGTGGAGGAGTACGCCTTCTTTAAGGACAGCGCCAAGATCGCTGATGAGCTGCGGCTGATCAAGTCCTTTGCCAGGATGGGAGAGCCAATCGCTGACGCTAGGAGAGCTATGTACATCGATGCCATCCGGATCCTGGGAACCAACCTGTCTTACGACGAGCTGAAGGCTCTGGCCGACACCTTCAGCCTGGATGAGAACGGCAACAAGCTGAAGAAGGGCAAGCACGGAATGCGCAACTTCATCATCAACAACGTGATCAGCAACAAGCGGTTTCACTACCTGATCAGATACGGCGACCCAGCTCACCTGCACGAGATCGCTAAGAACGAGGCCGTGGTGAAGTTCGTGCTGGGACGGATCGCCGATATCCAGAAGAAGCAGGGCCAGAACGGAAAGAACCAGATCGACCGCTACTACGAGACCTGCATCGGCAAGGATAAGGGAAAGTCCGTGTCTGAGAAGGTGGACGCTCTGACCAAGATCATCACAGGCATGAACTACGACCAGTTCGATAAGAAGAGATCTGTGATCGAGGACACCGGAAGGGAGAACGCCGAGAGAGAGAAGTTTAAGAAGATCATCAGCCTGTACCTGACAGTGATCTACCACATCCTGAAGAACATCGTGAACATCAACGCTAGATACGTGATCGGCTTCCACTGCGTGGAGCGCGATGCCCAGCTGTACAAGGAGAAGGGATACGACATCAACCTGAAGAAGCTGGAGGAGAAGGGCTTTAGCTCCGTGACCAAGCTGTGCGCTGGAATCGACGAGACAGCCCCCGACAAGAGGAAGGATGTGGAGAAGGAGATGGCCGAGAGAGCTAAGGAGAGCATCGACTCCCTGGAGTCTGCTAACCCTAAGCTGTACGCCAACTACATCAAGTACTCCGATGAGAAGAAGGCCGAGGAGTTCACCAGGCAGATCAACAGAGAGAAGGCCAAGACCGCTCTGAACGCCTACCTGAGGAACACAAAGTGGAACGTGATCATCCGGGAGGACCTGCTGCGCATCGATAACAAGACCTGTACACTGTTCCGGAACAAGGCTGTGCACCTGGAGGTGGCTCGCTACGTGCACGCCTACATCAACGACATCGCCGAGGTGAACTCCTACTTTCAGCTGTACCACTACATCATGCAGAGGATCATCATGAACGAGAGATACGAGAAGTCTAGCGGCAAGGTGTCTGAGTACTTCGACGCCGTGAACGATGAGAAGAAGTACAACGATAGACTGCTGAAGCTGCTGTGCGTGCCTTTCGGATACTGTATCCCACGGTTTAAGAACCTGAGCATCGAGGCCCTGTTCGACCGCAACGAGGCTGCCAAGTTTGATAAGGAGAAGAAGAAGGTGAGCGGCAACTCCGGTTCTGGTCTCGAGCCCAAGAAGAAGAGGAAAGTCGACTACAAGGACGACGATGACAAGTGATGACTCGAGCTGGTACTGCATGCACGCAATGCTAGCTGCCCCTTTCCCGTCCTGGGTACCCCGAGTCTCCCCCGACCTCGGGTCCCAGGTATGCTCCCACCTCCACCTGCCCCACTCACCACCTCTGCTAGTTCCAGACACCTCCCAAGCACGCAGCAATGCAGCTCAAAACGCTTAGCCTAGCCACACCCCCACGGGAAACAGCAGTGATTAACCTTTAGCAATAAACGAAAGTTTAACTAAGCTATACTAACCCCAGGGTTGGTCAATTTCGTGCCAGCCACACCCTGGAGCTAGCAAAAAAAAAAAAAAAAAAAAAAAAAAAAAAGCATATGACTAAAAAAAAAAAAAAAAAAAAAAAAAAAAAAAAAAAAAAAAAAAAAAAAAAAAAAAAAAAAAAAAAAAAAA
